# The Effect of Pharmaceutical Excipients on Protein Chemical Degradation Through Deamidation and Isomerization

**DOI:** 10.1007/s11095-026-04040-4

**Published:** 2026-03-13

**Authors:** Ingrid Ramm, Carl Diehl, Amanda Västberg, Johanna Hjalte, Herje Schagerlöf, Marie Wahlgren, Lars Nilsson

**Affiliations:** 1 Department of Process and Life Science Engineering, Lund University, 221 00 Lund, Sweden; 2 SARomics Biostructures AB, 223 63, Lund, Sweden; 3 Research Institutes of Sweden, 114 28 Stockholm, Sweden; 4 Global Research Technology, Novo Nordisk A/S, 2760 Måløv, Denmark; 5 MAX IV Laboratory, Lund, Sweden

**Keywords:** chemical stability, deamidation, liquid formulation, stabilizing excipients, therapeutic proteins

## Abstract

**Introduction:**

Therapeutic proteins are crucial in the treatment of a wide range of diseases. However, the proteins are sensitive to chemical degradation reactions, particularly deamidation and isomerization, which can compromise efficacy and safety. Formulation excipients, such as sugars and non-ionic surfactants, are commonly used to enhance stability, yet their effects on chemical degradation remain insufficiently understood.

**Methods:**

This study investigates how fructose, sucrose, melezitose, and the non-ionic surfactants polysorbate 80 and DDM (n-Dodecyl-β-D-maltoside) affect the structure and chemical stability of the affibody GA-Z, a protein prone to deamidation and isomerization. Chemical degradation and conformational changes were characterized using peptide fingerprinting, Liquid Chromatography-Mass Spectrometry, Titration fluorescence spectroscopy, two-dimensional Nuclear Magnetic Resonance spectroscopy, and Differential Scanning Calorimetry.

**Results:**

All three sugars lowered chemical degradation by stabilizing the folded state of the z-domain and inducing minor structural changes in the albumin-binding domain, thereby lowering the propensity for deamidation and isomerization. Polysorbate 80 showed minimal impact on both degradation and protein structure. In contrast, DDM increased deamidation and isomerization due to surfactant–protein interactions, resulting in structural changes.

**Conclusion:**

These results demonstrate how excipient-induced structural changes affect chemical degradation of proteins in liquid formulations. This study contributes to the understanding and design of more effective formulations for therapeutic proteins, enhancing their stability and safety.

**Graphical Abstract:**

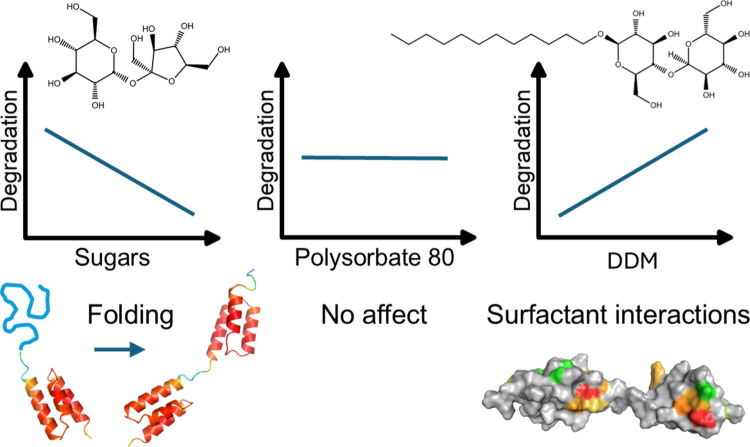

**Supplementary Information:**

The online version contains supplementary material available at 10.1007/s11095-026-04040-4.

## Introduction

Therapeutic proteins are widely used in modern medicine and are increasingly represented among the highest-earning pharmaceuticals. In 2024, five of the ten top-selling drugs worldwide were therapeutic proteins [1]. However, proteins are prone to both physical and chemical degradation in solution, which can reduce drug efficacy and, in some cases, trigger immunogenic responses [2–11]. To stabilize proteins and reduce denaturation, aggregation, deamidation, isomerization, oxidation, etc., excipients are added to liquid formulations.

Among chemical degradation reactions, deamidation and isomerization are the two most common [2, 3]. Their rates in solution are influenced by physical and chemical properties of the solvent and the protein itself, including, e.g., ionic strength, pH, dielectric constant, and the charge of nearby amino acid residues [12–21]. Deamidation and isomerization are initiated when a deprotonated peptide backbone amide nitrogen attacks the side chain carbonyl group of asparagine and aspartic acid residues [12]. The primary and higher-order structures can promote these reactions by bringing the reacting groups into close proximity [22]. Additionally, increased peptide backbone mobility increases the likelihood of conformations that shorten the distance between these groups, further enhancing the risk of the reactions [12, 13, 16, 17, 22, 23]. Conversely, secondary and tertiary structures can stabilize proteins by imposing conformational restrictions [18, 24–28]. Furthermore, the peptide backbone amide nitrogen is often involved in hydrogen bonding, in both α-helices and β-turns, which decreases its reactivity [29].

Sugars are often used to enhance the physical stability of therapeutic proteins. Sucrose is the most common sugar excipient and is typically included at concentrations of 25–200 mg/mL in liquid formulations [30, 31]. Sugars stabilize proteins against denaturation and aggregation [32–35], primarily through preferential exclusion from the protein surface, which drives the protein toward a folded state [36–39]. They have also been reported to increase enzyme stability and activity [35, 40]. Some studies have been conducted on the effect of sugars on deamidation and isomerization. Sucrose and trehalose lowered the deamidation and isomerization of denatured lysozyme [41] and sucrose and mannitol reduced the deamidation of peptides [33, 42, 43]. Additionally, a study on a protein in an optimized formulation showed that sucrose increased chemical degradation [44]. However, there is a lack of extensive systematic studies analyzing how sugars stabilize proteins from chemical degradation.

Non-ionic surfactants are primarily included in liquid formulations of therapeutic proteins to inhibit aggregation, increase solubility, and reduce surface adsorption. Polysorbate 80 is the most commonly used surfactant, with a critical micelle concentration of approximately 0.013 mM, and typical formulation concentrations of 0.01–2 mg/mL [30, 31]. Another non-ionic surfactant, n-Dodecyl-β-D-maltoside (DDM), is frequently used to solubilize membrane proteins [45] but is not used in therapeutic protein formulations [30, 31]. DDM has a critical micelle concentration of 0.17 mM in water [45]. Despite their widespread use, the effect of surfactants on chemical stability, particularly deamidation and isomerization, remains poorly understood.

This study aims to investigate how different excipients affect the structure and chemical stability of the affibody GA-Z, which is known to degrade through deamidation and isomerization [46]. Three sugars of varying size, fructose, sucrose, and melezitose, and the two non-ionic surfactants, polysorbate 80 and DDM, were added to GA-Z in solution. The chemical stability and the effects of the excipients on degradation were evaluated using peptide fingerprinting and liquid chromatography-mass spectrometry (LC–MS). Structural changes were assessed by titration fluorescence spectroscopy and two-dimensional NMR spectroscopy, while thermal stability was characterized using differential scanning calorimetry (DSC).

## Materials and Methods

### Materials

The affibody GA-Z was provided by Swedish Orphan Biovitrum AB (Stockholm, Sweden) at a stock concentration of 90 mg/mL in a 25 mM sodium phosphate buffer with 125 mM NaCl at pH 7.0 and was stored at − 80°C before use. GA-Z is a small two-domain protein with a molecular weight of 11 900 Da and an isoelectric point (pI) of approximately 4.5. Fructose (≥ 99%), sucrose (≥ 99.5%), melezitose (≥ 99.0%), tris-hydrochloride (Molecular Biology grade, ≥ 99%), urea (BioUltra, ≥ 99%), and trypsin (Porcine, MS grade) were purchased from Sigma-Aldrich (MO, USA). Sodium phosphate dibasic dihydrate (Na_2_HPO_4_•2H_2_O, 99–102%) and sodium phosphate dibasic monohydrate (NaH_2_PO_4_•H_2_O, 98.5–100.5%) were obtained from Merck (NJ, USA). Formic acid (LC–MS graded, 99%) and sodium chloride (100%) were purchased from VWR (PA, USA). Polysorbate 80 (Super Refined) was provided by Croda (UK), and n-Dodecyl-β-D-maltoside (analytical grade) was purchased from Anatrace Products (OH, USA). Acetonitrile (Optima LC–MS) and formic acid (Optima LC–MS) were purchased from Fisher Chemical (NH, USA). Milli-Q grade water (Merck Millipore) was used for all buffers, mobile phases, and sample preparations.

### Accelerated Stability Study and Sample Preparation

Stability study samples contained 9 mg/mL GA-Z in 25 mM sodium phosphate buffer (Merck) with 125 mM NaCl (VWR) adjusted to pH 7.0, together with 0–30% v/v fructose, sucrose, or melezitose (Sigma), 0–10.9 mM DDM (Anatrace Products), or 0–0.8 mM polysorbate 80 (Croda). Samples were prepared in duplicate and incubated at 37°C for 0–44 days to promote degradation. All sodium phosphate-buffered saline (PBS) solutions with added excipients were autoclaved prior to use to prevent bacterial growth during storage. Furthermore, samples were prepared under sterile conditions and stored at − 80°C after incubation until further analysis.

### Calculations of Sugar Volumes

The volume fractions of sugar were calculated using Eq. 1.

1 $${v}_{sugar}/{v}_{total}\,\left(\%\right)=\left(\frac{{n}_{sugar}*{M}_{w, sugar}}{{\rho \left(g/l\right)}_{sugar}}/{v}_{total}\right)*100$$

where n_sugar_ is the mol of sugar, M_w,sugar_ is the molecular weight of sugar, ρ(g/l)_sugar_ is the density of sugar in water, and v_total_ is the total volume. The density ρ(g/l)_sugar_ was calculated using Eq. 2:

2 $${\rho (g/l)}_{sugar}=\frac{{wt (g/g)}_{sugar}}{\frac{1}{{\rho (g/l)}_{sugar+water}}-\frac{1-{wt (g/g)}_{sugar}}{{\rho (g/l)}_{water}}}$$

where ρ(g/l)_sugar+water_ is the density of the sugar-water solution and ρ(g/l)_water_ is the density of water. Both ρ(g/l)_sugar+water_ and ρ(g/l)_water_ are experimental values from the literature [47, 48]. The sugar mass fraction wt(g/g)_sugar_ was calculated using the Eq. (3).

3 $${wt\, (g/g)}_{sugar}=\frac{{n}_{sugar}*{M}_{w, sugar}}{{\rho (g/l)}_{sugar+water}*{v}_{total}}$$

### Water Activity

The water activity of samples containing 9 mg/mL GA-Z in PBS buffer (25 mM sodium phosphate, 125 mM NaCl, pH 7.0) with 0–30% v/v fructose, sucrose, or melezitose, 0–0.8 mM polysorbate 80, or 0–10.9 mM DDM, was measured at 20°C using an AquaLab system (Decagon Devices, WA, USA). The instrument was calibrated with pure water (aw = 1) and two AquaLab reference standards with water activities of 0.250 and 0.760 (Decagon Devices, WA, USA). All measurements were performed in triplicate.

### Trypsin Digestion of GA-Z

To enable the identification and quantification of degraded residues, the stability samples were subjected to trypsin digestion. For samples containing DDM or polysorbate 80, the surfactants were removed prior to digestion using Standard Line Screw-Cap Microcentrifuge Tubes (VWR) following the manufacturer’s instructions. For denaturation, 5 µL of each sample (corresponding to 45 µg GA-Z) was mixed with 85 µL of 8 M urea (Sigma-Aldrich) in 50 mM tris-hydrochloride buffer, pH 7.4 (Sigma-Aldrich), and incubated for 10 min at 37°C. Since GA-Z does not contain disulfide bonds, reduction and alkylation were not required. The urea concentration was then diluted to < 1 M by adding 650 μL of 50 mM tris-hydrochloride buffer (pH 7.4). Trypsin (0.8 μg, Sigma-Aldrich) was added to achieve a trypsin to protein ratio of 1:56 (w/w), and the samples were digested for 3 h at 37°C. The digestion was stopped by adding 4 μL of formic acid (VWR) to a final concentration of > 0.5% v/v. The samples were then stored at − 80°C for up to 7 days before LC–MS analysis.

### Liquid Chromatography-Mass Spectrometry (LC–MS)

The LC–MS analysis was performed on an Agilent 1290 Infinity II system (Agilent Technologies, CA, USA) coupled to an Agilent 6550 Q-TOF Ion Funnel mass spectrometer (Agilent Technologies) using an XBridge BEH C18 XP column (130 Å, 2.5 µm, 3 × 150 mm, Waters, MA, USA). UV detection was carried out at 280 nm, and samples were kept at 4°C in the autosampler. Mobile phase A consisted of 0.1% formic acid (Fisher Chemical) in Milli-Q water, and mobile phase B consisted of 0.1% formic acid (Fisher Chemical) in acetonitrile (Honeywell). The gradient was held at 5% B for 0–5 min, increased to 55% B from 5 to 40 min, and then to 95% B from 40 to 44 min. The flow rate was 0.3 mL/min, the injection volume was 10 µL, and the column oven temperature was 40°C. During the 5–40 min interval, the LC flow was directed to the MS source.

The mass spectrometer was operated in positive ion mode with a mass range of 100–2000 m/z, acquiring data at a rate of 5 spectra per second. The Dual AJS ESI source conditions were as follows: drying gas temperature 290°C, drying gas flow 14 L/min, sheath gas temperature 225°C, sheath gas flow 12 L/min, nebulizer pressure 55 psi, capillary voltage 4500 V, nozzle voltage 2000 V, fragmentor voltage 300 V, skimmer voltage 65 V, and octupole RF voltage 750 V. Data processing was performed using the MassHunter software (Agilent Technologies).

### Differential Scanning Calorimetry (DSC)

The thermal stability of GA-Z in the presence and absence of fructose, sucrose, melezitose, DDM, or polysorbate 80 was investigated using DSC. The samples contained 9 mg/mL GA-Z in PBS buffer (25 mM sodium phosphate, 125 mM NaCl, pH 7.0), and were analyzed in duplicate. The measurements were performed on a micro-PEAQ cal-DSC instrument (Malvern, Worcestershire, UK) by increasing the temperature from 10 to 100°C at a rate of 1°C/min. Medium thermal feedback setting and three purge refills were applied. Data analysis was carried out using the MicroCal PEAK-DSC software (Malvern) with a spline function for peak fitting and buffer subtraction. The thermograms were normalized to the protein concentrations.

### Titration Fluorescence Spectroscopy

Titration fluorescence spectroscopy was conducted to analyze structural changes in GA-Z induced by DDM or polysorbate 80. The measurements were performed on a Probe Drum (Labbot, Sweden), measuring intrinsic protein fluorescence. Experiments were carried out at 20 and 37°C. Excitation was set to 280 nm, and emission spectra were recorded from 240 to 760 nm. Samples contained 9 mg/mL GA-Z in PBS (25 mM sodium phosphate, 125 mM NaCl, pH 7.0) with an initial volume of 600 μL. Titrations were performed using polysorbate 80 (7.6 mM) or DDM (117.5 mM). Every 71 s, 2 μL of the titrant was added to reach final concentrations of 0–1.0 mM polysorbate 80 and 0–15.3 mM DDM. Following each addition, the sample was equilibrated for 60 s before measurement, and mixing was applied at the speed setting 4 (of 7). All measurements were conducted in duplicate.

### Nuclear Magnetic Resonance (NMR) Spectroscopy

Structural changes in GA-Z induced by fructose, sucrose, melezitose, DDM, or polysorbate 80 were examined using two-dimensional NMR spectroscopy. ^1^H-^13^C SOFAST-HMQC spectra were acquired at the Swedish NMR centre at Gothenburg University, Sweden, on a Bruker Advance III HD 800 MHz spectrometer equipped with a 5 mm TXO cold probe. All measurements were conducted at 37°C. The ^1^H spectra width was set to 13.22 ppm and the ^13^C spectral width to 30 ppm, with 1280 and 128 points in the direct and indirect dimensions, respectively. Spectra were processed in the nmrPipe software suite [49] using solvent filter in the direct dimension, squared cosine apodization, and zero-filling in both dimensions, followed by baseline correction in the direct dimension. Processed spectra were visualized and analyzed using the CCPNMR software suite [50]. Additionally, ^1^H spectra were recorded using 126 scans, an acquisition time of 2.048 s, a relaxation delay of 1.5 s, and a spectral width of 16 kHz.

## Results

**Fig. 1:**
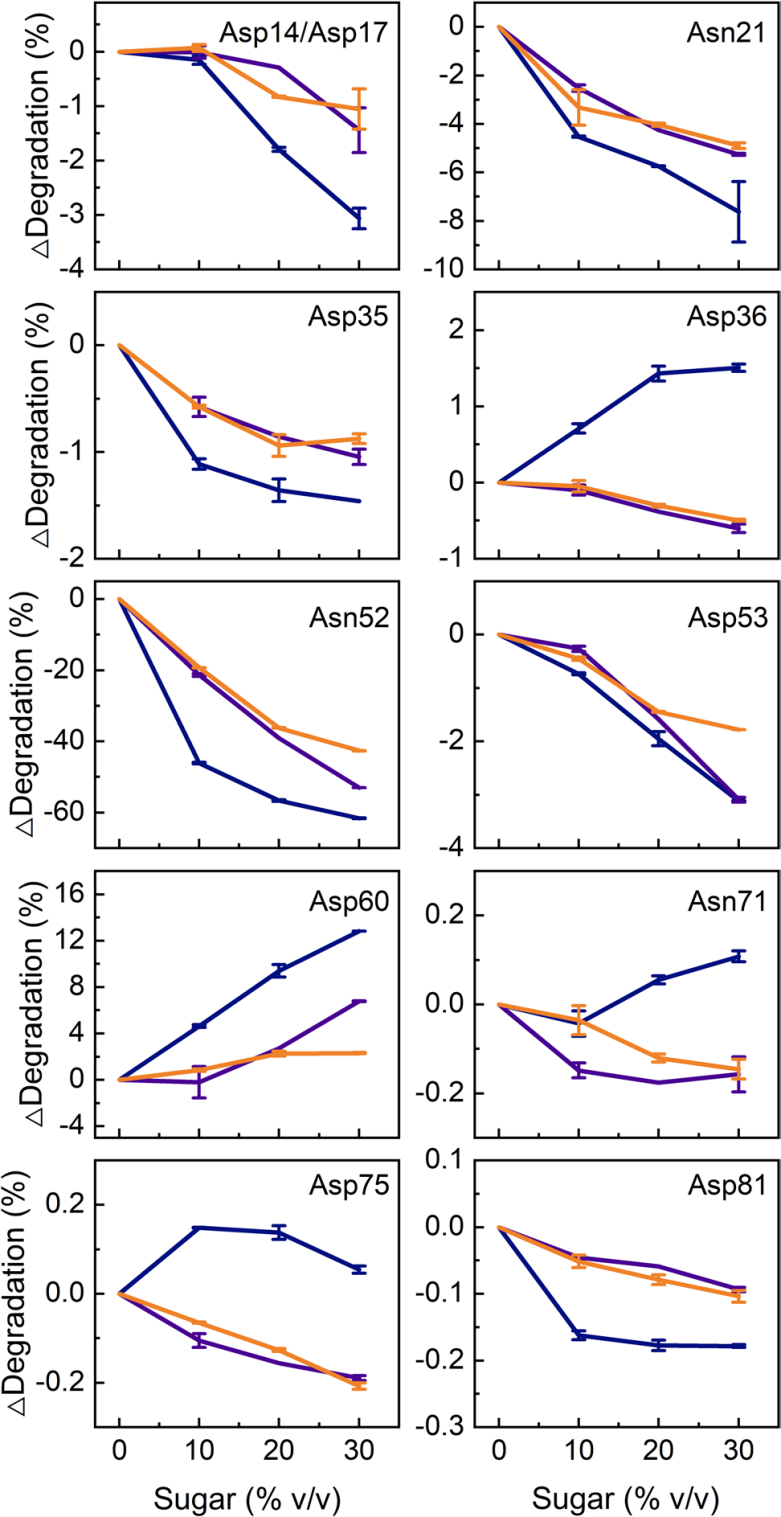
Change in deamidation or isomerization of individual GA-Z residues vs. sugar concentration (% v/v) after 44 days of incubation at 37°C in fructose (  ), sucrose (  ), or melezitose (  ) as determined by LC–MS. All samples contained 9 mg/mL GA-Z in PBS buffer (25 mM sodium phosphate, 125 mM NaCl, pH 7.0) and were prepared in duplicate. Error bars represent the standard deviation between duplicates.

**Fig. 2:**
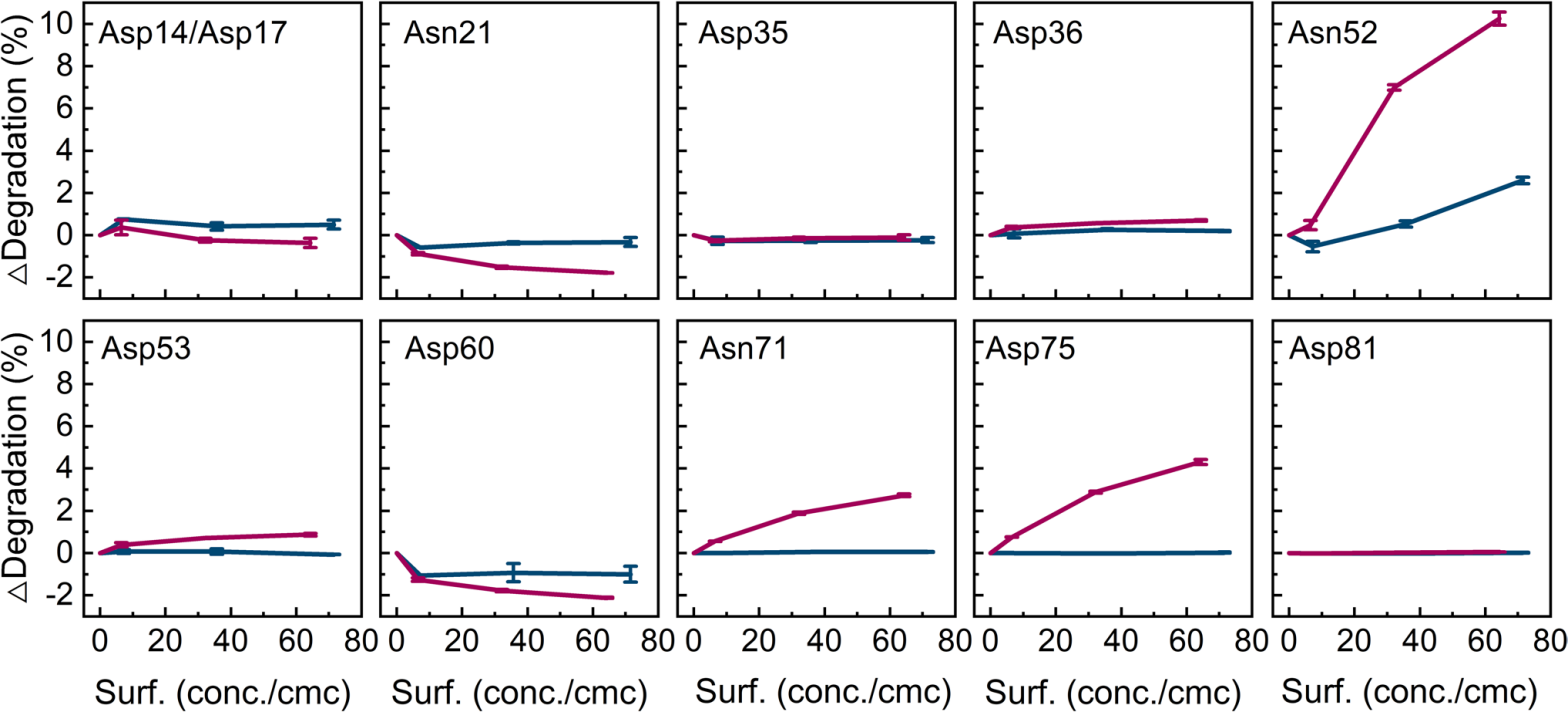
Change in deamidation or isomerization of individual GA-Z residues vs. surfactant concentration, normalized against its corresponding critical micelle concentration (c/cmc), after 44 days of incubation at 37°C in DDM (  ) and polysorbate 80 (  ) as determined by LC–MS. All samples contained 9 mg/mL GA-Z in PBS buffer (25 mM sodium phosphate, 125 mM NaCl, pH 7.0) and were prepared in duplicate. Error bars represent the standard deviation between duplicates.

In this study, the effects of three different sugars and two non-ionic surfactants on the deamidation and isomerization of GA-Z after storage at 37°C were analyzed using LC–MS (Figs. 1 and 2). Fructose, sucrose, and melezitose all reduce degradation (Fig. 1). Fructose, however, affects the chemical degradation of the residues somewhat differently than sucrose and melezitose. Because fructose is a reducing sugar, GA-Z underwent chemical reactions with fructose during storage, as evidenced by visible color changes in the samples after a few days of incubation. These reactions may have altered the GA-Z peptide compositions, potentially affecting their LC–MS retention times and, consequently, reducing the accuracy of their quantified degradation amounts.

Sucrose and melezitose provide comparable stabilizing effects on GA-Z when the reduction in degradation was evaluated against the sugar volume fractions. Both sugars stabilize asparagine residues at all positions, and aspartic acid residues found in α-helices and loops connecting α-helices. In contrast, isomerization of Asp60, located in the linker connecting the two domains, increases with rising sugar concentration (0–30% v/v). The positions of these residues have been reported previously [46]. Consistent with previous findings [46], most of the chemical degradation occurred within the z-domain.

The chemical stability of GA-Z in the presence of DDM and polysorbate 80 was evaluated by LC–MS (Fig. 2). The surfactant concentrations were normalized to the critical micelle concentration (CMC) of the respective surfactant. Polysorbate 80 has minimal effect on the chemical stability, with only Asn52 in α-helix 3 of the z-domain showing slight destabilization, and no other asparagine or aspartic acid residues being affected. In contrast, DDM increases the degradation of the residues Asn71 and Asp75 in α-helix 1 of the albumin-binding domain, Asn52 and Asp53 in α-helix 3 of the z-domain, while reducing the degradation of Asn21 in the loop connecting α-helix 1 and 2 of the z-domain and Asp60 in the linker.

**Fig. 3:**
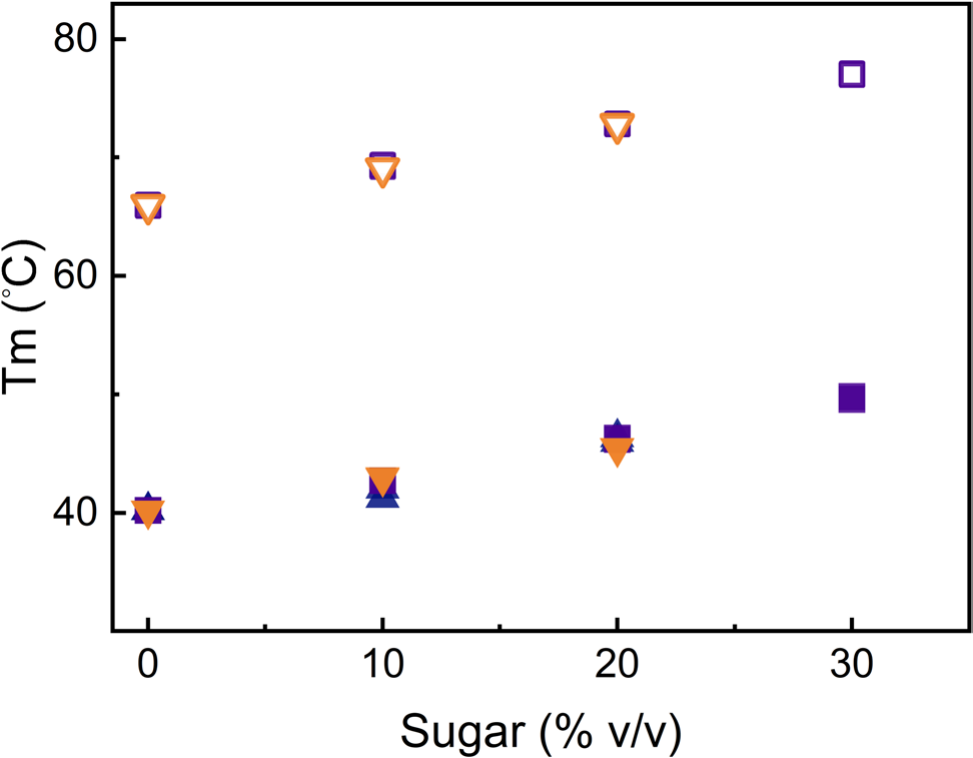
Denaturation temperatures of GA-Z as determined by DSC. Tm_1_ of the z-domain vs. sugar concentration (% v/v) for fructose (  ), sucrose (  ), or melezitose (  ) and Tm_2_ of the albumin-binding domain vs. sugar concentration (% v/v) for sucrose (  ) and melezitose (  ). All samples contained 9 mg/mL GA-Z in PBS buffer (25 mM sodium phosphate, 125 mM NaCl, pH 7.0). Measurements were performed in duplicate, and the data are included in the figure.

The denaturation temperatures (Tm) of GA-Z in 0–30% v/v fructose, sucrose, or melezitose are shown in Fig. 3, with corresponding thermograms in Figs. S1−3 (Supplementary Information). Denaturation temperatures could not be obtained for 30% v/v melezitose, since it is insoluble below 20°C, or 30% v/v fructose, because fructose undergoes chemical reactions at higher temperatures. For fructose, Tm_2_ was not determined, as the denaturation of GA-Z was only analyzed up to 53°C due to the interfering chemical reactions of the reducing sugar.

A previous study has shown that GA-Z exhibits two denaturation temperatures corresponding to its two domains, Tm_1_ for the z-domain and Tm_2_ for the albumin-binding domain [46]. All three sugars increase the Tm_1_ and Tm_2_ linearly at the investigated concentration range. The sugar-induced increase of Tm_1_ reduced the amount of unfolded z-domain during storage at 37°C. The albumin-binding domain, however, was in its folded state at all sugar concentrations, as Tm_2_ is much higher than 37°C. Because all sugars induced comparable increases in Tm_1_ and Tm_2_, it is concluded that the sugar-induced increase in thermal stability of GA-Z is directly proportional to the sugar volume fractions.

**Fig. 4:**
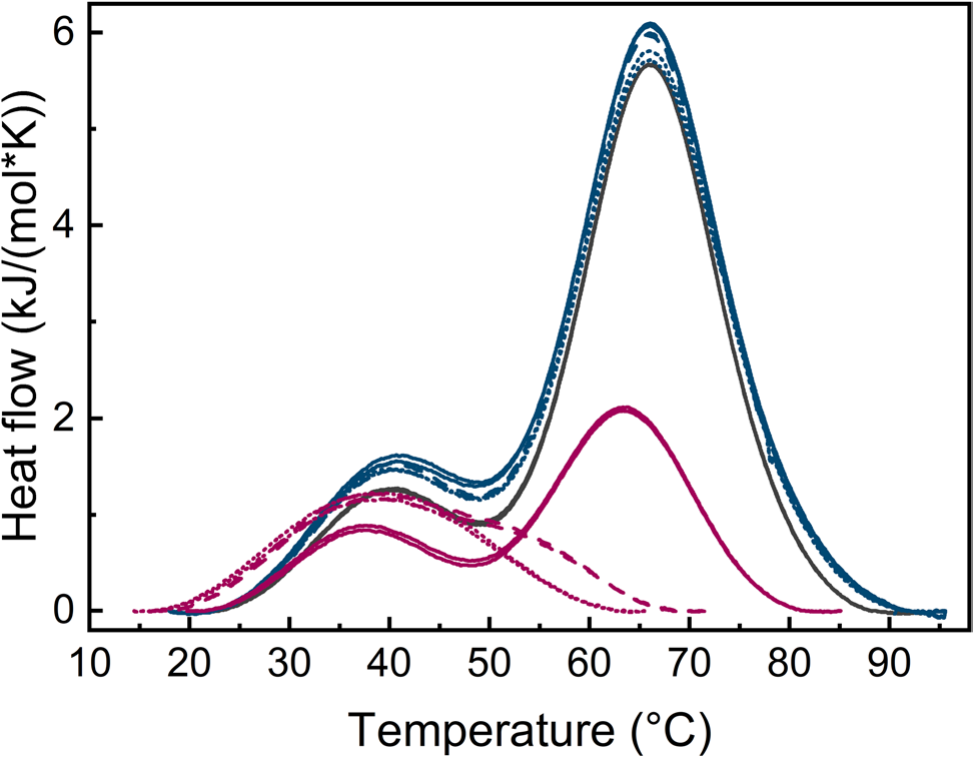
DSC thermograms of GA-Z in the presence of DDM (0–10.9 mM) and polysorbate 80 (0–0.8 mM). Thermograms for DDM are shown at 0 (—), 1.1 (  ), 5.5 (  ), and 10.9 (  ) mM DDM, corresponding to 0, 6, 32, and 64 c/cmc, respectively. Thermograms for polysorbate 80 are shown at 0.1 (  ), 0.4 (  ), and 0.8 (  ) mM polysorbate 80, corresponding to 7, 36, and 71 c/cmc, respectively. All samples contained 9 mg/mL GA-Z in PBS buffer (25 mM sodium phosphate, 125 mM NaCl, pH 7.0). Measurements were performed in duplicate, and the data are included in the figure.

DSC measurements were also performed on GA-Z in the presence of the two surfactants DDM and polysorbate 80, at concentrations ranging from 0–10.9 mM DDM and 0–0.8 mM polysorbate 80. The resulting thermograms are shown in Fig. 4. The addition of polysorbate 80 does not change the positions of the DSC peaks, indicating that Tm_1_ and Tm_2_ remain unchanged for all investigated concentrations. These results suggest that polysorbate 80 does not affect the thermal stability of GA-Z. For DDM, however, increasing the concentrations of DDM caused the second peak (Tm_2_) to gradually disappear. This observation shows that DDM strongly affects the thermal stability of GA-Z and may indicate that the albumin-binding domain loses its structure upon addition of DDM.

**Fig. 5:**
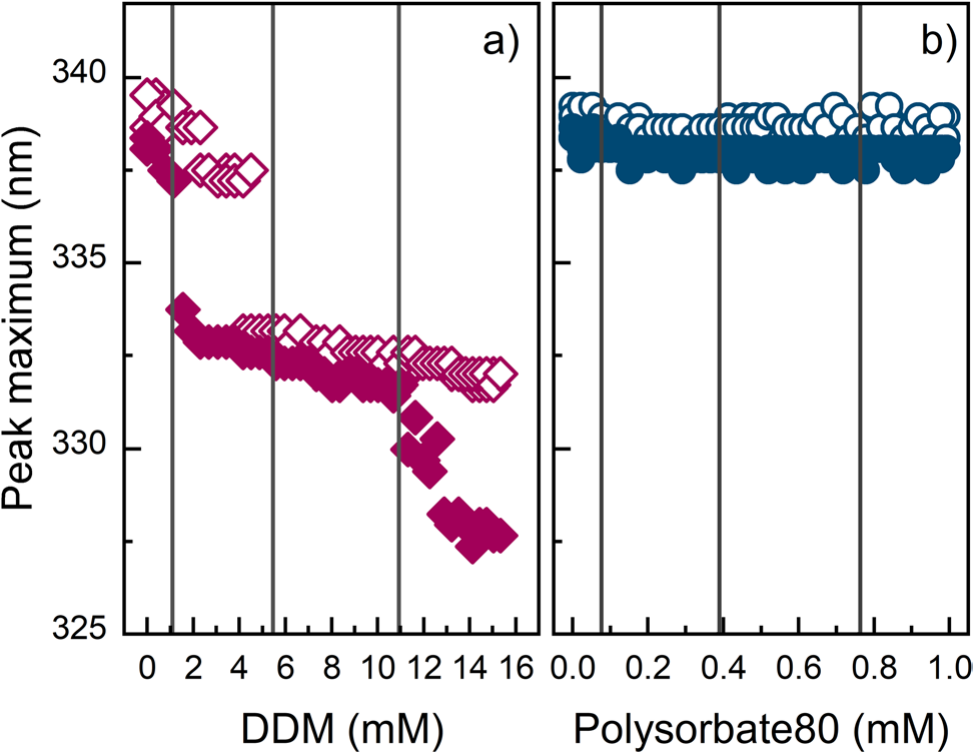
Titration fluorescence spectroscopy of GA-Z in the presence of DDM and polysorbate 80. Emission peak maximum of GA-Z vs surfactant molar concentrations of **a**) DDM at 20°C (  ) and 37°C (  ), and **b**) polysorbate 80 at 20°C (  ) and 37°C (  ). Black lines represent the concentrations of DDM and polysorbate 80 used in the stability studies. All samples contained 9 mg/mL GA-Z in PBS buffer (25 mM sodium phosphate, 125 mM NaCl, pH 7.0). Measurements were performed in duplicate, and the data are included in the figure.

To further investigate how the surfactants affect the GA-Z structure, titration fluorescence spectroscopy was performed with DDM or polysorbate 80. The surfactants were titrated into GA-Z solutions, generating concentration ranges of 0–15.3 mM DDM and 0–1.0 mM polysorbate 80. Figure 5 shows the wavelength at the emission peak maximum at increasing polysorbate 80 and DDM concentrations. The measurements were conducted at 20 and 37°C.

The titrations with polysorbate 80 show no indication of structural changes in GA-Z at either temperature (Fig. 5b). These experiments were performed at a lower total surfactant concentration compared to DDM, as the titrant amounts were normalized to the surfactant’s critical micelle concentration (CMC). An additional experiment at 20°C with a polysorbate 80 concentration endpoint of 15.3 mM confirmed that even at this substantially higher concentration, polysorbate 80 did not affect GA-Z structure (data not shown).

In contrast, DDM induces a pronounced blue shift of the emission peak maximum (Fig. 5a), indicating that the environments of the aromatic residues are becoming more hydrophobic [51]. At 20°C and 0 mM DDM, the emission peak maximum is 340 nm, and a decrease from 338 to 333 nm occurs between 3.8 and 4.2 mM DDM. At 37°C, the emission peak maximum is initially lower, 338 nm, followed by a decrease from 337 to 333 nm between 1.2 and 1.5 mM DDM, and a second decrease from 332 to 328 nm between 11.0 and 12.9 mM DDM.

**Fig. 6:**
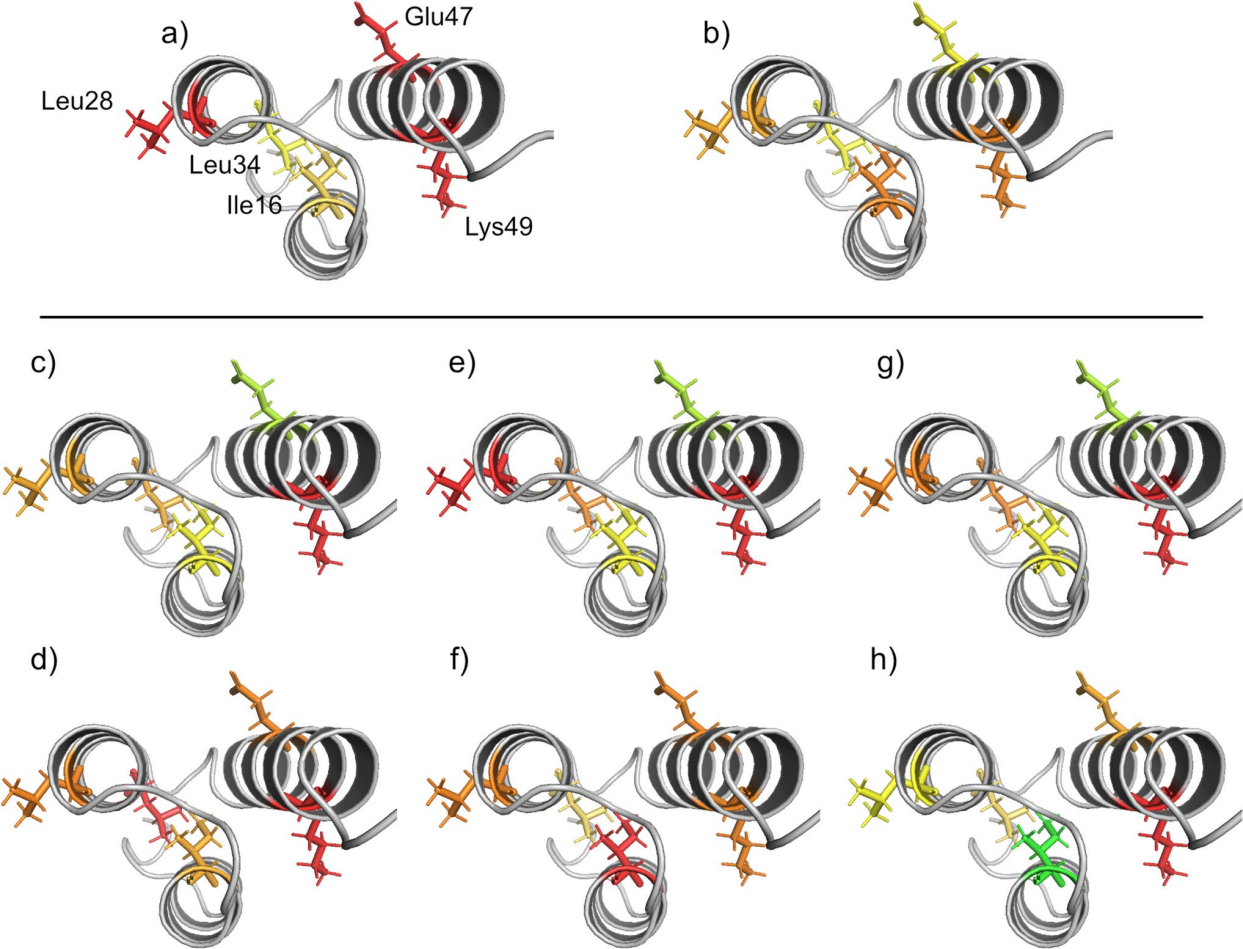
Effect of temperature [46] and sugars on HMQC peak intensities and chemical shifts of assigned residues in the z-domain. **a**-**b**) changes upon decreasing the temperature from 42 to 22°C, and changes upon the addition of 30% v/v **c**-**d**) fructose, **e**–**f**) sucrose, and **g**-**h**) melezitose at 37°C. Color coding indicates the magnitude of changes, where green to yellow represents lower changes, and orange to red represents higher changes. All samples contained 9 mg/mL GA-Z in PBS buffer (25 mM sodium phosphate, 125 mM NaCl, pH 7.0). HMQC spectra of GA-Z in the presence of 0 and 20% v/v sugars are shown in Fig. S4, and HMQC peak intensities and chemical shifts at increasing sugar concentrations are provided in Figs. S5-10 and Table S1.

Sugar-induced conformational changes in GA-Z were investigated using the two-dimensional NMR experiment SOFAST-HMQC. Spectra were acquired at 37°C, and 0–30% v/v fructose, sucrose, or melezitose were added to GA-Z. Figure 6 illustrates how the sugar addition and a temperature decrease from 42°C to 22°C affect the HMQC peak intensities and chemical shifts of assigned residues in the z-domain of GA-Z. The AlphaFold2 structure of GA-Z, peak assignments, and the temperature-dependent data were obtained from a previous study [46]. Residue-specific changes are color-coded, with larger changes in intensity or chemical shift shown in orange to red, and smaller changes in green to yellow. In the absence of sugars, approximately 50% of the population of GA-Z exists in an unfolded state at 37°C, as the denaturation temperature of the z-domain is 40°C (Fig. 3). The presence of sugars increases the denaturation temperature, thereby stabilizing the folded state. At 22°C, the z-domain is fully folded, as denaturation begins at 26°C (Figs. S1−3). An increase in the HMQC peak intensity, observed either upon sugar addition or temperature reduction, indicates that the local environment of the corresponding residue shifts toward a more ordered state during the stabilization of the folded state [46].

Lowering the temperature causes the environments of surface-exposed residues, Glu47, Leu28, and Lys49, to shift toward a more ordered state, as shown by their red coloration. In contrast, the residues in the core, Leu34 and Ile16, are less affected. All three sugars affect the z-domain in a similar manner, where the environments of Leu28, Leu34, and Lys49 shift toward a more ordered state (orange to red), and Ile16 and Glu47 exhibit little to no change. These results suggest that reducing the temperature to 22°C shifts the surface-exposed residues toward a more ordered state more effectively than the sugar addition. This difference may reflect incomplete folding of the z-domain in the presence of 30% v/v sugars at 37°C (Figs. S1−3).

When the folded state of the z-domain is stabilized by lowering the temperature, residues exhibit small to larger chemical shift changes (yellow to orange). These changes arise from alterations in their local chemical environments and are likely due to minor structural rearrangements. The addition of fructose induces larger chemical shift changes across all residues (orange to red), suggesting that fructose causes a minor overall structural change in the z-domain. This indicates that the presence of fructose has a larger impact on the structure of GA-Z than temperature reduction. Similarly, sucrose induces chemical shift changes in most residues, except for Ile16, located in the core of the domain. Like fructose, sucrose likely induces an overall structural change. Melezitose, however, causes larger chemical shift changes for the residues Glu47 and Lys49, both located in α-helix 3, while residues in α-helices 1 and 2, Leu28, Leu34, and Ile16, undergo smaller changes.

**Fig. 7:**
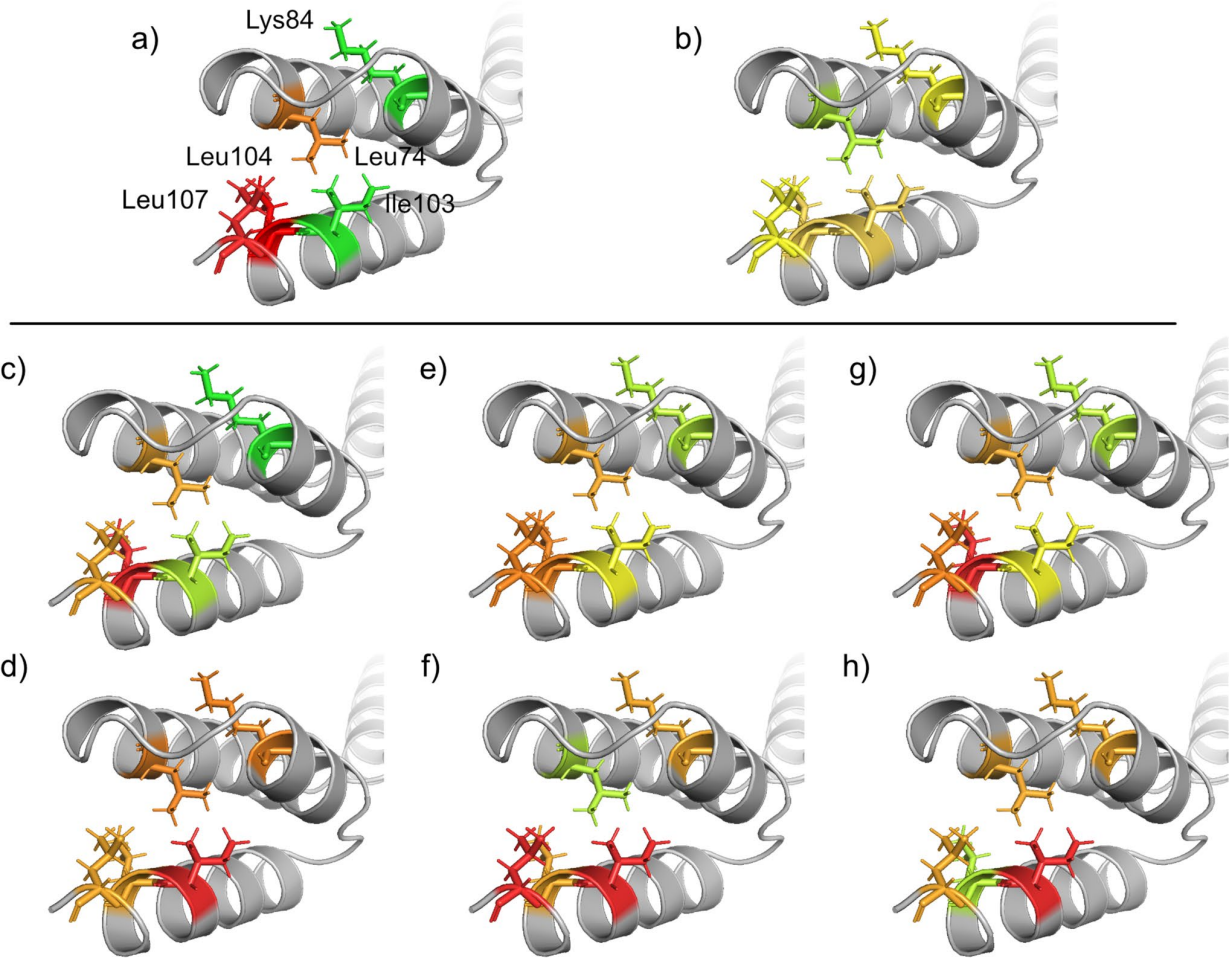
Effect of temperature [46] and sugars on HMQC peak intensities and chemical shifts of assigned residues in the albumin-binding domain. **a-b)** changes upon decreasing the temperature from 42 to 22°C, and changes upon the addition of 30% v/v **c-d)** fructose, **e–f)** sucrose, and **g-h)** melezitose at 37°C. Color coding indicates the magnitude of changes, where green to yellow represents lower changes, and orange to red represents higher changes. All samples contained 9 mg/mL GA-Z in PBS buffer (25 mM sodium phosphate, 125 mM NaCl, pH 7.0). HMQC spectra of GA-Z in the presence of 0 and 20% v/v sugars are shown in Fig. S4, and HMQC peak intensities and chemical shifts at increasing sugar concentrations are provided in Figs. S5-10 and Table S1.

Figure 7 shows how the presence of 30% v/v fructose, sucrose, or melezitose, as well as a temperature reduction from 37 to 22°C, affects the HMQC peak intensities and chemical shifts of assigned residues in the albumin-binding domain. Similar to the z-domain, all three sugars comparably influence the albumin-binding domain. Despite the albumin-binding domain being in a folded state at 37°C (Figs. S1−3), the presence of sugar causes the environments of Leu74, Leu104, and Leu107 to shift toward a more ordered state, as shown by their coloration (orange to red). Other residues in the domain remain unaffected (green to yellow). Figure 7a shows that the temperature reduction also shifts the environments of Leu74, Leu104, and Leu107 toward a more ordered state. A previous study hypothesized that this shift results from an extension of α-helix 3 to incorporate parts of the C-terminal loop [46].

Notably, the temperature reduction does not induce chemical shift changes in the albumin-binding domain, whereas all three sugars do. Fructose, sucrose, and melezitose alter the chemical shifts of all assigned residues, with the exceptions of Leu74 (in the presence of sucrose) and Leu104 (in the presence of melezitose). These sugar-induced chemical shift changes suggest that the sugars induce small structural changes in the albumin binding domain that are not attributable to stabilization of the folded state.

**Fig. 8:**
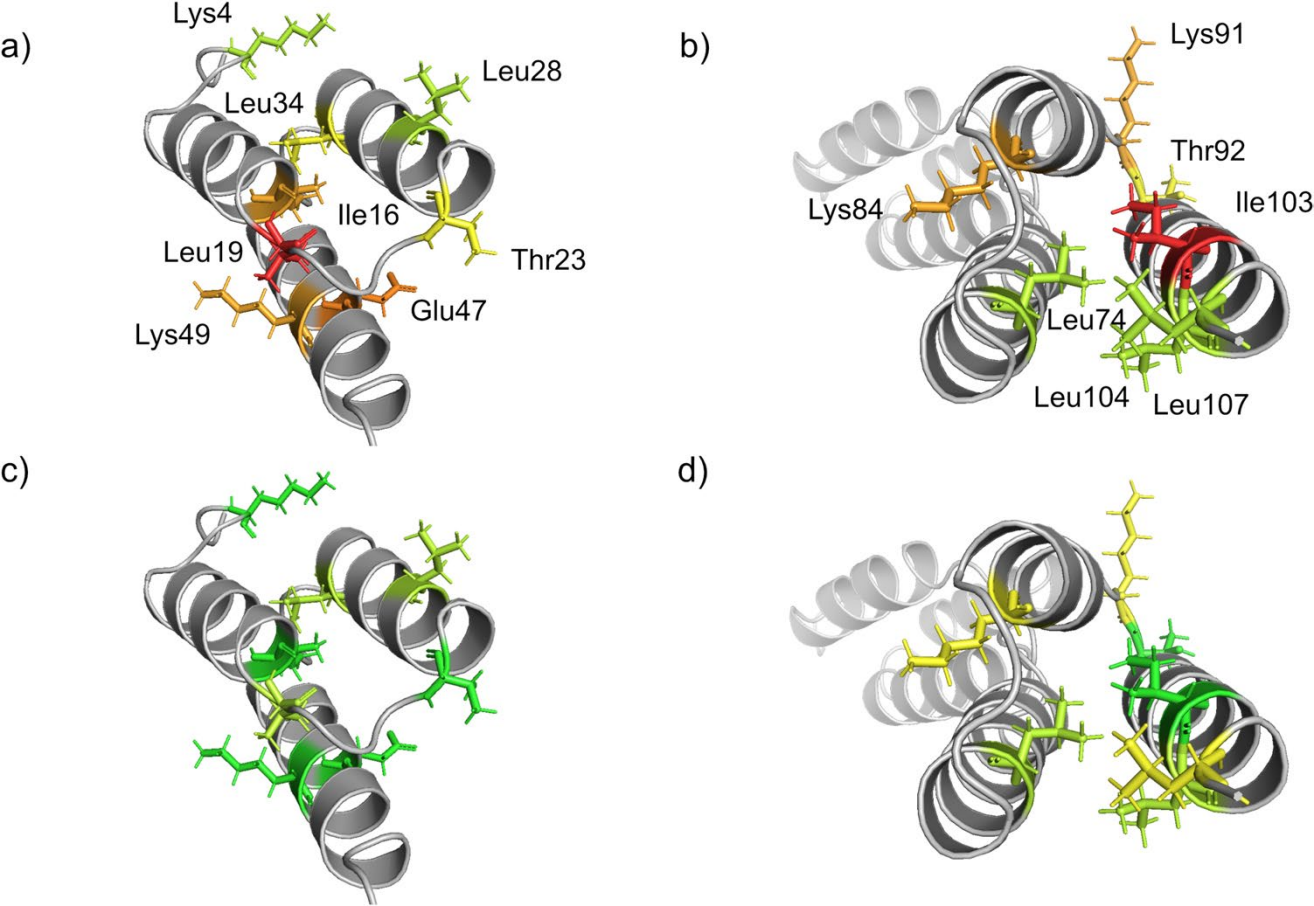
Effect of surfactants on HMQC chemical shifts of assigned residues in GA-Z. Changes upon the addition of **a-b)** 10.9 mM DDM and **c-d)** 0.8 mM polysorbate 80 at 37°C. Color coding indicates the magnitude of changes, where green to yellow represents lower changes, and orange to red represents higher changes. All samples contained 9 mg/mL GA-Z in PBS buffer (25 mM sodium phosphate, 125 mM NaCl, pH 7.0). HMQC spectra of GA-Z in the presence of 0 and 10.9 mM DDM and 0.8 mM polysorbate 80 are shown in Fig. S4, and chemical shifts at increasing surfactant concentrations are provided in Table S2.

Structural changes induced by DDM and polysorbate 80 were monitored using the two-dimensional NMR experiment SOFAST-HMQC. Spectra were acquired at 37°C across concentrations of 0–10.9 mM DDM and 0–0.8 mM polysorbate 80. Figure 8 shows how surfactants affect the chemical shifts of assigned residues in GA-Z. Polysorbate 80 induces only minimal chemical shift changes across all residues, within the range of experimental error, as indicated by their coloration (green to yellow). In contrast, DDM induces larger shift changes (orange to red) for Ile16, Leu19, Glu47, and Lys49 in the z-domain and Lys84, Lys91, and Ile103 in the albumin-binding domain. These changes show that DDM alters the chemical environment of these residues, likely due to structural changes in the residues and/or their surroundings.

DDM both increases and decreases the chemical stability of GA-Z (Fig. 2). Increased degradation may result from a loss of ordered structure, such as loss of secondary structure or intramolecular hydrogen bonding, or a shift to a conformation with a higher propensity for deamidation and isomerization, characterized by a shorter Cγ-N distance. Conversely, stabilization is associated with an increase in ordered structure or an increased Cγ-N distance [18, 22, 24, 25, 28, 29, 46]. Thus, changes in GA-Z chemical stability induced by DDM indicate conformational changes at the affected residues.

**Fig. 9:**
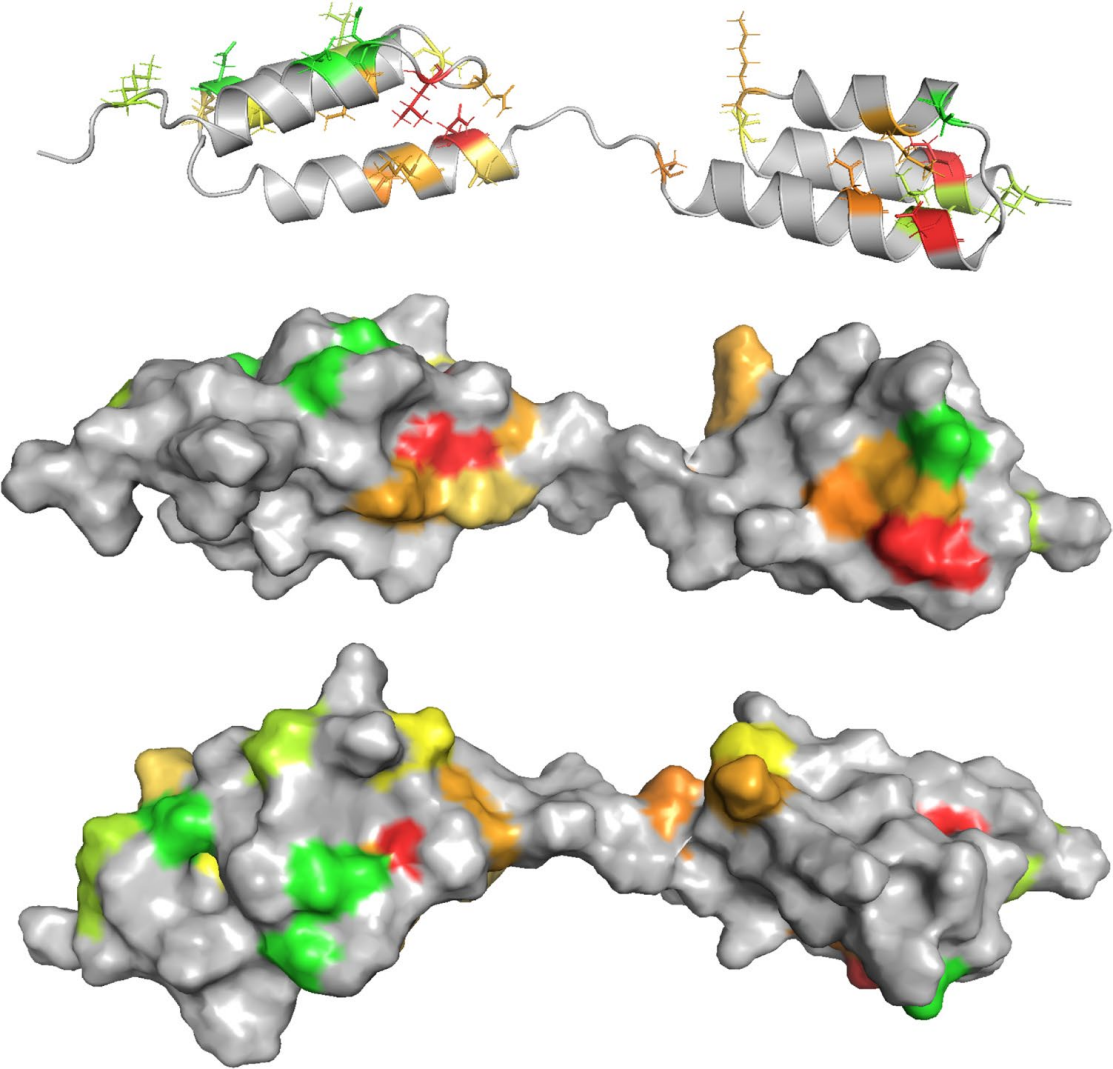
DDM-induced changes in GA-Z upon addition of 0–10.9 mM DDM at 37°C. Color coding indicates the magnitude of changes in chemical stability and chemical shifts, where green to yellow represents low changes, and orange to red represents high changes. All samples contained 9 mg/mL GA-Z in PBS buffer (25 mM sodium phosphate, 125 mM NaCl, pH 7.0).

By combining the chemical degradation data and the NMR chemical shift changes, a structural map of DDM-induced alterations was generated (Fig. 9). Small changes are colored from green to yellow, and large changes from orange to red. In both domains, orange/red regions are observed where several residues show DDM-induced alterations. In the z-domain, the orange/red region includes the core of the z-domain, the loop connecting α-helix 1 and 2, and α-helix 3. The linker is likely involved as well, given the proximity of this area and the observed change in Asp60. In the albumin-binding domain, an orange/red area includes residues in α-helix 1, α-helix 2, and α-helix 3.

Titration fluorescence spectroscopy of GA-Z revealed changes in the environment of aromatic residues upon DDM addition. Only two aromatic residues are located near the orange/red regions identified in the structural map. Trp27 in α-helix 2 lies at the edge of the orange/red area in the z-domain, and Tyr83 is positioned spatially close to Ile103 and adjacent to Lys84 in α-helix 2 in the albumin-binding domain. These observations suggest that conformational changes of Trp27 and Tyr83 are likely responsible for the shifts seen in the fluorescence emission spectra (Fig. 5).

## Discussion

All asparagine and aspartic acid residues of GA-Z, except for Asp60 in the linker, exhibit increased chemical stability when adding sucrose and melezitose. The sugars also enhance the thermal stability of GA-Z by increasing the denaturation temperatures of both domains. The ability of sugar to stabilize proteins against denaturation has been reported previously [32, 35]. Experimental and simulation studies have demonstrated the preferential exclusion of solutes from protein surfaces [35, 36, 52–56], and the stabilization of GA-Z by sugars is most likely caused by this mechanism. Interestingly, the sugar-induced increase in the denaturation temperatures of GA-Z is proportional to the sugar volume fractions, suggesting that the stabilization against denaturation caused by the exclusion depends on the volume of the excluded sugar molecules. Previous studies have concluded that the increased thermal stability of proteins depends on the size of the stabilizing solute [32, 56]. However, these studies have compared the solutes at the same molar concentration. A larger size generates a larger volume fraction at a given molar concentration and thus provides increased stabilization due to a larger excluded volume.

Figure 1 shows that sucrose and melezitose exert similar effects on the chemical stability of residues within the z-domain, with this stabilization correlating with the sugar volume fractions. It is, therefore, likely that the chemical stability of the z-domain, as well as thermal stability, depends on the preferential exclusion of sugars. At 37°C, increasing sugar volume fractions shift the z-domain toward a folded state (Fig. 6, Figs. S1−3). The shift toward a folded state decreases the conformational flexibility of the peptide backbone, which is known to lower the propensity for deamidation and isomerization [18, 22, 24, 25, 28, 29]. Thus, the reduced chemical degradation observed in the z-domain is most likely a consequence of the sugar-induced stabilization of its folded state. Together, these findings demonstrate that the chemical stability of the z-domain is enhanced by stabilizing the folded state induced through the preferential exclusion of sugars.

Figure 1 shows that the residues in the albumin-binding domain, like those in the z-domain, are stabilized to a similar extent by sucrose and melezitose, indicating that their chemical stability depends on the sugar volume fractions and may also depend on the preferential exclusion of sugar. Because this domain is in a folded state at 37°C (Fig. 6, Figs. S1−3), the increased chemical stability cannot be explained by a sugar-induced stabilization of the folded state. However, the NMR data reveal that the sugars induce small structural changes in the albumin-binding domain (Fig. 7), similar to the previously reported effects of 30% v/v glycerol, which were associated with a shift of the structure toward a more ordered state [46]. If the structural changes observed in the albumin-binding domain correlate with a shift toward a more ordered state, the structural changes would lower the conformational flexibility of the peptide backbone and thereby enhance the chemical stability.

Sucrose and melezitose stabilize the aspartic acid residues located in loops. However, glycerol has been shown to destabilize these residues, which was attributed to a lowering of the dielectric constant of the solvent [46]. A reduced dielectric constant increases the pK_a_ of the aspartic acid carboxylic side chain, making it a better leaving group and thereby promoting isomerization [12]. Sugars also lower the dielectric constant of the solvent [57], and a similar destabilizing effect would therefore be expected. Indeed, fructose and sucrose destabilize Asp60 in the linker, most likely due to the lowered dielectric constant.

The observed stabilization of loop-located aspartic acid residues by sucrose and melezitose suggests that these sugars impose structural restrictions on the peptide backbone, as increased stability is often associated with reduced conformational flexibility [18, 22, 24, 25, 28, 29]. As stated, the NMR data indicate that α-helix 3 in the albumin-binding domain is extended upon addition of fructose, sucrose, and melezitose. The stabilized aspartic acid residues are positioned adjacent to α-helices, and it is possible that introducing the sugars extends nearby α-helices to incorporate these residues. Helical incorporation would reduce backbone flexibility and thereby reduce isomerization. Although this interpretation aligns with the observed stabilization, the lack of NMR assignments within the loops prevents definitive confirmation.

Polysorbate 80 has a minimal effect on the chemical stability of GA-Z (Fig. 2), except for a slight destabilization of Asn52. In addition, polysorbate 80 does not affect the thermal stability or the structure of GA-Z (Figs. 4, 5, and 8), nor does it markedly change solvent properties, such as water activity (Figs. S11), which could potentially influence the chemical stability [8, 12, 58]. The absence of changes in chemical degradation indicates that polysorbate 80 does not interact substantially with GA-Z.

DDM changes the chemical stability of GA-Z and has a pronounced effect on both the thermal stability and protein structure (Figs. 2, 4, 5, and 8). These changes in chemical stability are likely a consequence of the DDM-induced alterations in thermal stability and structure, as protein conformation is well known to influence degradation rates [18, 22, 24, 25, 28, 29]. The combined observations strongly indicate that DDM interacts directly with GA-Z. As no interaction between polysorbate 80 and GA-Z is observed, hydrophobicity is likely not the driving force for DDM/GA-Z interaction. Rather, the interaction could originate from the maltose head-group of DDM. Some proteins are known to bind carbohydrates, such as sugars [59, 60]. These interactions are not fully understood but are known to often involve π-interaction between aromatic residues and sugars, with tryptophan being the most abundant residue at sugar–protein binding sites [59, 60]. Asparagine and aspartic acid residues are also common at these sites [59, 60]. Hence, DDM surfactants may associate with regions of GA-Z where asparagine, aspartic acid, or aromatic residues are positioned. Moreover, the observed blue shift in the fluorescence emission indicates that the environment surrounding the tryptophan residue(s) of GA-Z becomes more hydrophobic upon DDM addition [51], and may be the result of DDM association.

Figure 9 shows that DDM affects residues in both domains of GA-Z as well as within the linker. Interestingly, the affected residues are positioned on the same side of the protein, suggesting that DDM predominantly interacts with one side of GA-Z. A possible explanation is that the protein interacts with a DDM micelle, forming a protein-micelle complex. The location of the altered residues could indicate that the formed complex is a decorated micelle, where the protein is oriented such that it is primarily one side that is in contact with the palisade layer of the DDM micelle [61–63].

Upon DDM addition, the DSC thermograms show that the transition peak corresponding to the albumin-binding domain (Tm_2_) disappears (Fig. 4). While this might suggest that the domain losses its structure, the NMR data show no evidence of a global loss of secondary or tertiary structure (Fig. 8). Thus, it is more likely that the possible formation of a decorated micelle decreases the conformational freedom of GA-Z, leading to the disappearance of Tm_2._

When increasing the DDM concentration, the stability and structural changes in GA-Z occur in steps (Figs. 4 and 5). Based on these observations, it is proposed that the following occurs at 37°C: Between 0–1.2 mM DDM, DDM unimers associate via their maltose head-groups to GA-Z. Between 1.5–11.0 mM DDM, more DDM unimers bind to GA-Z, and the increasing number of DDM molecules interacting with GA-Z leads to the formation of a decorated micelle including both domains.

As described, DDM both increases and decreases the chemical stability of specific residues in GA-Z. Residues that exhibit increased degradation likely adopt structures that enhance their propensity for deamidation and isomerization. These structural changes may increase the conformational flexibility of the peptide backbone or lower the Cγ-N distances. Specifically, residues positioned in α-helix 3 in the z-domain and in α-helix 1 in the albumin-binding domain are destabilized by DDM. This destabilization may result from the loss of their α-helix structures upon DDM binding, which would increase the conformational flexibility of their peptide backbone. Additionally, DDM-induced structural changes could reduce the Cγ-N distances of these residues. Conversely, two residues situated in loops are stabilized by DDM. The formation of a decorated micelle may decrease the conformational flexibility of the loops where these residues reside, thereby reducing their degradation.

## Conclusions

This study provides important insights into how the excipients fructose, sucrose, melezitose, DDM, and polysorbate 80 influence the chemical stability of GA-Z. The presence of the sugars enhances the stability of GA-Z by lowering deamidation and isomerization and increasing its denaturation temperatures. The increase in the denaturation temperature of the z-domain stabilizes the folded state of the domain. Additionally, the sugars induce small structural changes in the albumin-binding domain, likely reducing its conformational flexibility. The stabilization of the folded state of the z-domain and the structural alterations of the albumin-binding domain shift the local structures of the degrading residues toward conformations with a lower propensity for deamidation and isomerization, thereby reducing degradation. This sugar-induced stabilization is likely closely linked to the preferential exclusion of sugars from the protein surface, with the effect being proportional to the sugar volume fractions. Polysorbate 80 shows minimal impact on the stability of the protein and its structure. In contrast, DDM strongly affects both the chemical stability and structure of GA-Z, most likely through interaction with the protein, driven by the maltose head-group of DDM. The findings suggest that these interactions alter the conformation of the protein, potentially forming a protein-micelle complex that modifies its stability. Overall, these results contribute to a deeper understanding of how different stabilizing excipients, such as sugars and surfactants, modulate protein stability, with implications for protein formulation and biopharmaceutical development.

## Supplementary Information

Below is the link to the electronic supplementary material.

Supplementary file1 (PDF 862 KB)

## Data Availability

The datasets generated during and/or analyzed during the current study are available from the corresponding author on reasonable request.

## Abbreviations

*DDM*: N-Dodecyl-β-D-maltoside
*DSC*: Differential Scanning Calorimetry
*LC-MS*: Liquid Chromatography-Mass Spectrometry
*NMR*: Nuclear Magnetic Resonance
*PBS*: Phosphate-buffered saline
*Tm*: Denaturation temperature
