# Supplementary material for: The Effect of Pharmaceutical Excipients on Protein Chemical Degradation Through Deamidation and Isomerization
Source: Pharm Res. 2026 Mar 13;43(4):1227–40. doi: 10.1007/s11095-026-04040-4 (PMC13179256; doi:10.1007/s11095-026-04040-4)
Supplement: Supplementary file 1 — Supplementary file1 (PDF 862 KB) [file 11095_2026_4040_MOESM1_ESM.pdf]

Supplementary Information - The effect of pharmaceutical excipients on protein chemical degradation through deamidation and isomerization

Ingrid Ramm[1], Carl Diehl[2], Amanda Västberg[1,3], Johanna Hjalte[1], Herje Schagerlöf[1,4], Marie Wahlgren[1], Lars Nilsson[1,5,*]

[1]Department of Process and Life Science Engineering, Lund University, 221 00 Lund, Sweden

[2]SARomics Biostructures AB, 223 63 Lund, Sweden

[3]Research Institutes of Sweden, 114 28 Stockholm, Sweden

[4]Global Research Technology, Novo Nordisk A/S, 2760 Måløv, Denmark

[5] MAX IV Laboratory, Lund, Sweden

*lars.nilsson@ple.lth.se

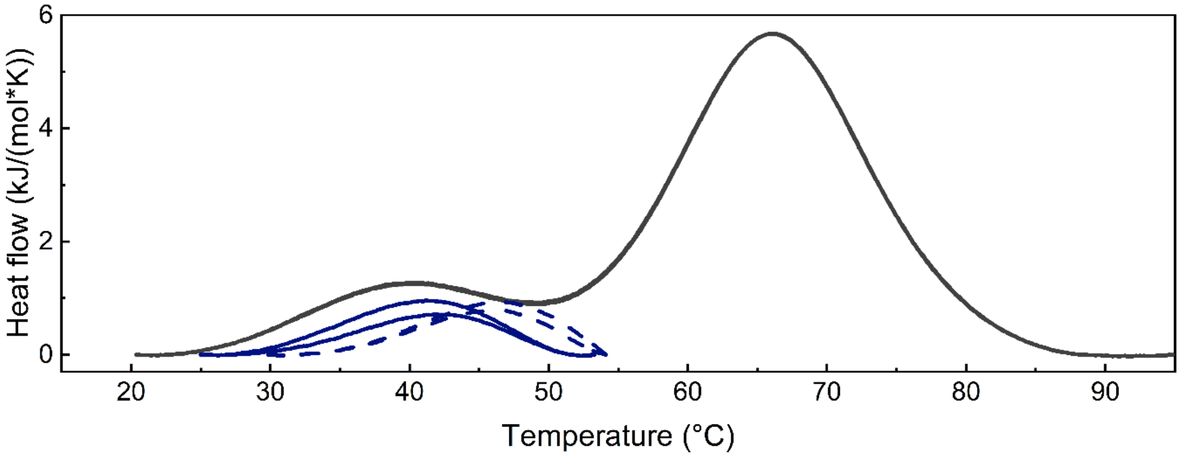

Fig. S1 DSC thermograms of GA-Z in 0 (—), 10 (—), and 20 (---) % v/v fructose. All samples contained 9 mg/mL GA-Z in PBS buffer (25 mM sodium phosphate, 125 mM NaCl, pH 7.0). Measurements were performed in duplicate, and the data are included in the figure

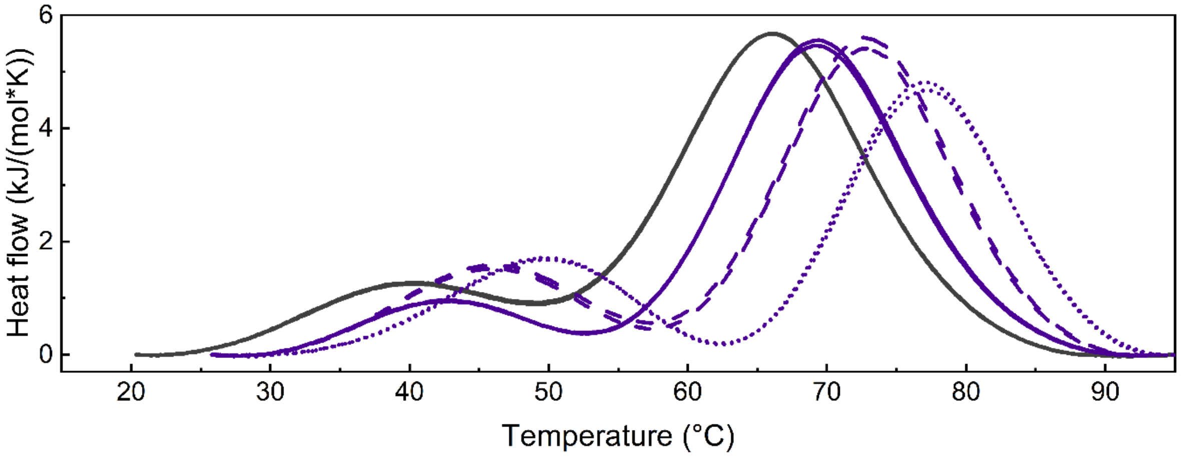

Fig. S2 DSC thermograms of GA-Z in 0 (—), 10 (—), 20 (---), and 30 (···) % v/v sucrose. All samples contained 9 mg/mL GA-Z in PBS buffer (25 mM sodium phosphate, 125 mM NaCl, pH 7.0). Measurements were performed in duplicate, and the data are included in the figure

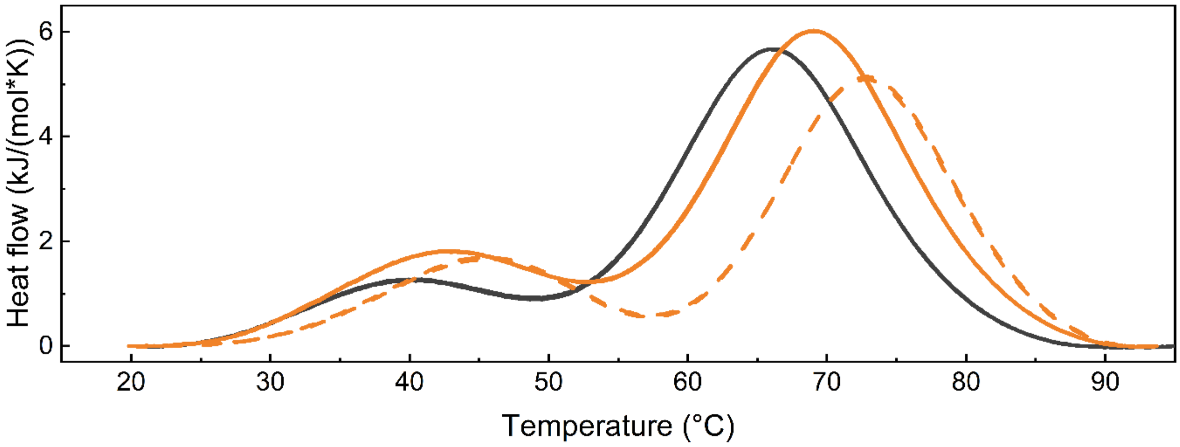

Fig. S3 DSC thermograms of GA-Z in 0 (—), 10 (—), and 20 (---) % v/v melezitose. All samples contained 9 mg/mL GA-Z in PBS buffer (25 mM sodium phosphate, 125 mM NaCl, pH 7.0)**.** Measurements were performed in duplicate, and the data are included in the figure

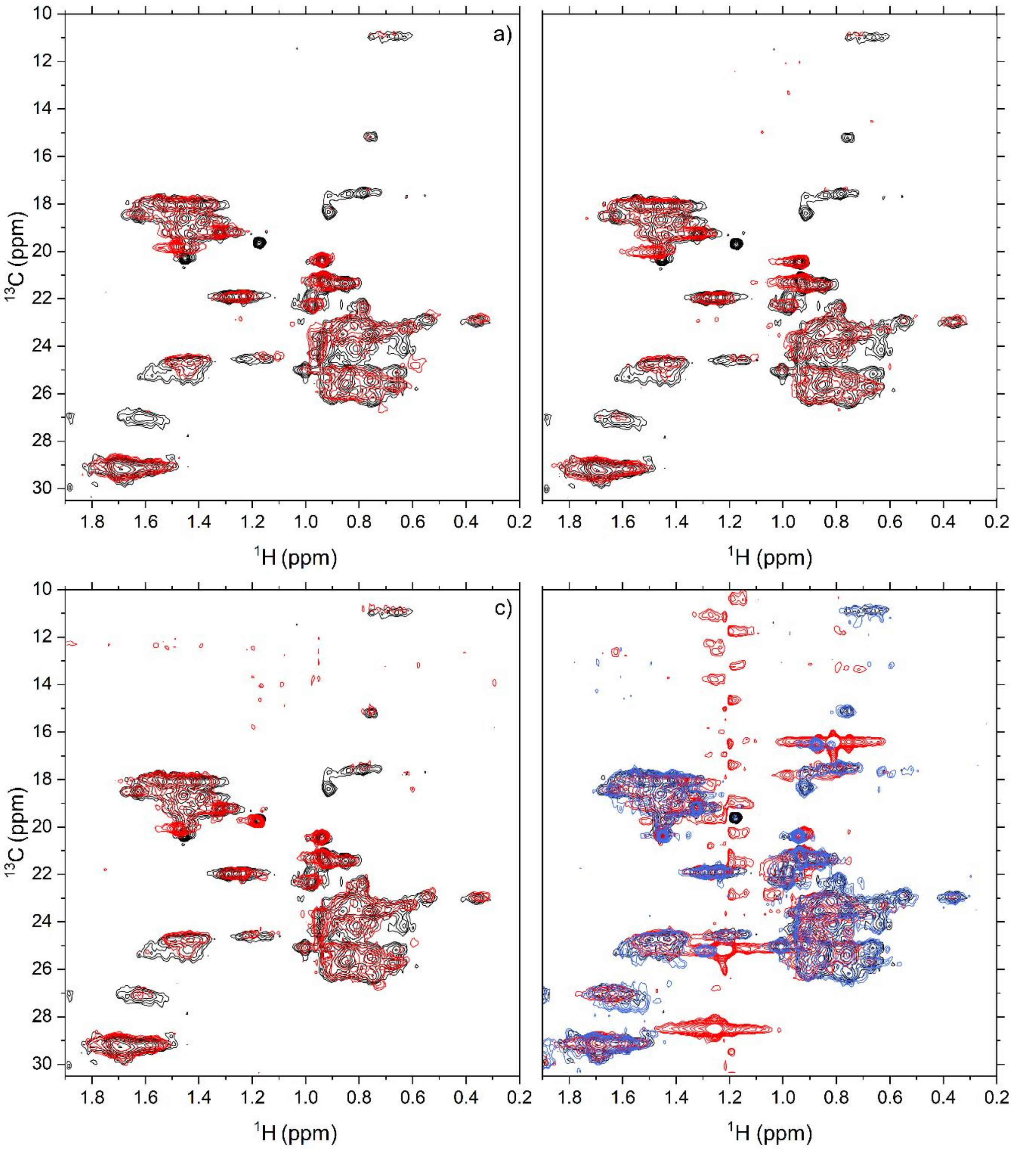

Fig. S4 HMQC spectra of GA-Z in a) 0 (—) and 20 (—) % v/v fructose, b) 0 (—) and 20 (—) % v/v sucrose, c) 0 (—) and 20 (—) % v/v melezitose, and d) 0 mM surfactant (—), 10.9 mM DDM (—), and 0.8 mM polysorbate 80 (—). All samples contained 9 mg/mL GA-Z in PBS buffer (25 mM sodium phosphate, 125 mM NaCl, pH 7.0), and the spectra were collected at 37°C

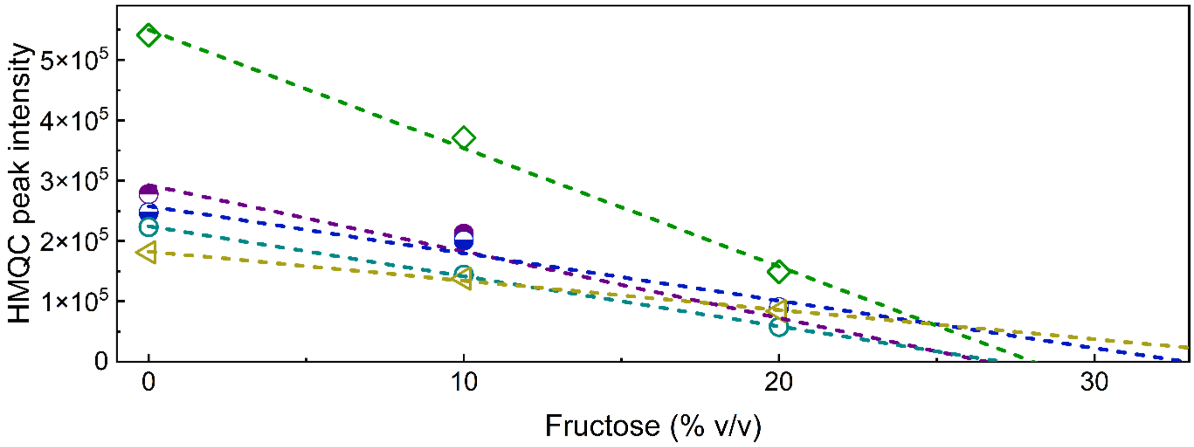

Fig. S5 HMQC peak intensities of the GA-Z residues Ile16$^{(C\gamma2\text{-}H\gamma2)}$ (◓), Leu28$^{(C\gamma\text{-}H\gamma)}$ (◒), Leu34$^{(C\gamma\text{-}H\gamma)}$ (○), Glu47$^{(C\beta\text{-}H\beta)}$ (◇), and Lys49$^{(C\gamma\text{-}H\gamma)}$ (◁) in 0-20% v/v fructose analyzed at 37°C. The slope equations for the residues are I=-11013*X+292803 (---), I=-7835*X+257891 (---), I=-8286.9*X+224533 (---), I=-19609*X+550046 (---), and I=-4839.3*X+182757 (---). All samples contained 9 mg/mL GA-Z in PBS buffer (25 mM sodium phosphate, 125 mM NaCl, pH 7.0)

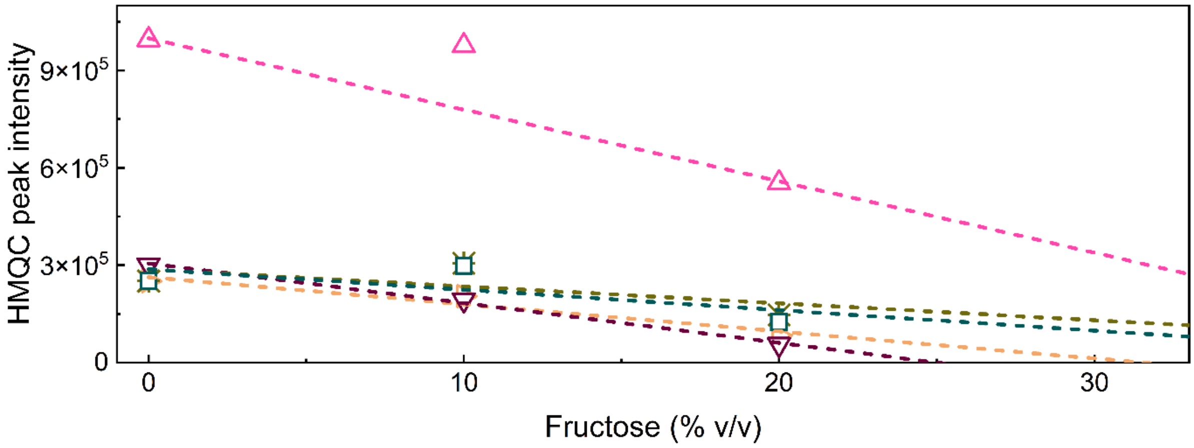

Fig. S6 HMQC peak intensities of the GA-Z residues Leu74$^{(C\delta2\text{-}H\delta2)}$ (▷), Lys84$^{(C\delta\text{-}H\delta)}$ (△), Ile103$^{(C\gamma2\text{-}H\gamma2)}$ (▽), Leu104 $^{(C\delta2\text{-}H\delta2)}$ (*), and Leu107$^{(C\delta2\text{-}H\delta2)}$ (□) in 0-20% v/v fructose analyzed at 37°C. The slope equations for the residues are I=-8363.6*X+263518 (---), I=-22043*X+1000000 (---), I=-12242*X+305975 (---), I= 5219.4*X+287160 (---), and I=-6307.2*X+288019 (---). All samples contained 9 mg/mL GA-Z in PBS buffer (25 mM sodium phosphate, 125 mM NaCl, pH 7.0)

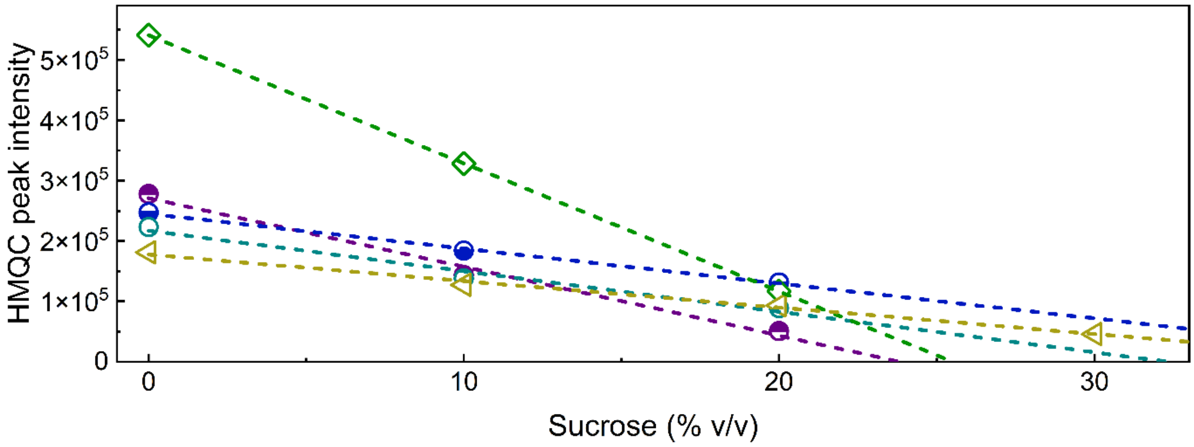

Fig. S7 HMQC peak intensities of the GA-Z residues Ile16$^{(C\gamma2\text{-}H\gamma2)}$ (◓), Leu28$^{(C\gamma\text{-}H\gamma)}$ (◒), Leu34$^{(C\gamma\text{-}H\gamma)}$ (○), Glu47$^{(C\beta\text{-}H\beta)}$ (◇), and Lys49$^{(C\gamma\text{-}H\gamma)}$ (◁) in 0-30% v/v sucrose analyzed at 37°C. The slope equations for the residues are I=-11372*X+271132 (---), I=-5783.9*X+245385 (---), I=-6717.5*X+217275 (---), I=-21214*X+540984 (---), and I=-4386.7*X+177756 (---). All samples contained 9 mg/mL GA-Z in PBS buffer (25 mM sodium phosphate, 125 mM NaCl, pH 7.0)

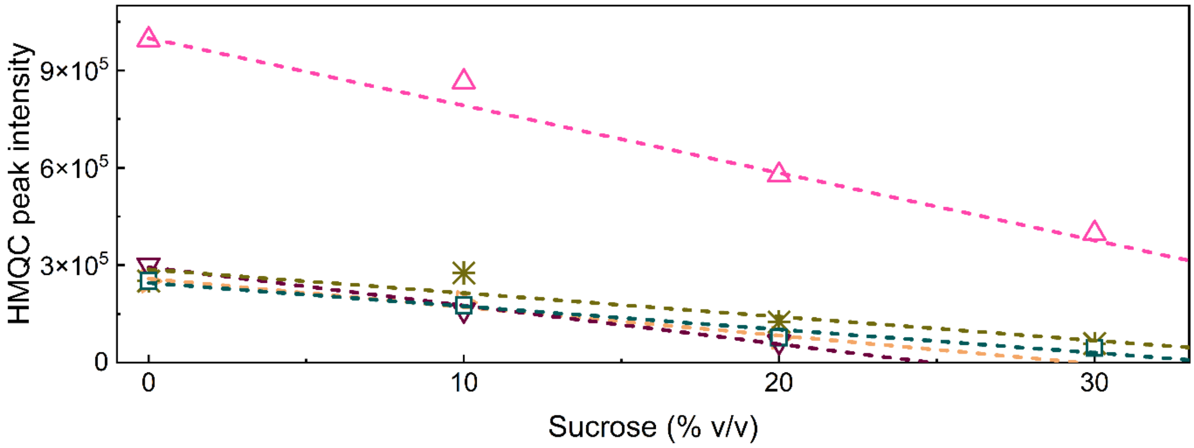

Fig. S8 HMQC peak intensities of the GA-Z residues Leu74$^{(C\delta2\text{-}H\delta2)}$ (▷), Lys84$^{(C\delta\text{-}H\delta)}$ (△), Ile103$^{(C\gamma2\text{-}H\gamma2)}$ (▽), Leu104 $^{(C\delta2\text{-}H\delta2)}$ (*), and Leu107$^{(C\delta2\text{-}H\delta2)}$ (□) in 0-30% v/v sucrose analyzed at 37°C. The slope equations for the residues are I=-8752.9*X+258537 (---), I=-20770*X+1000000 (---), I=-11845*X+294049 (---), I=-7296.5*X+287314 (---), and I=-7175*X+245456 (---). All samples contained 9 mg/mL GA-Z in PBS buffer (25 mM sodium phosphate, 125 mM NaCl, pH 7.0)

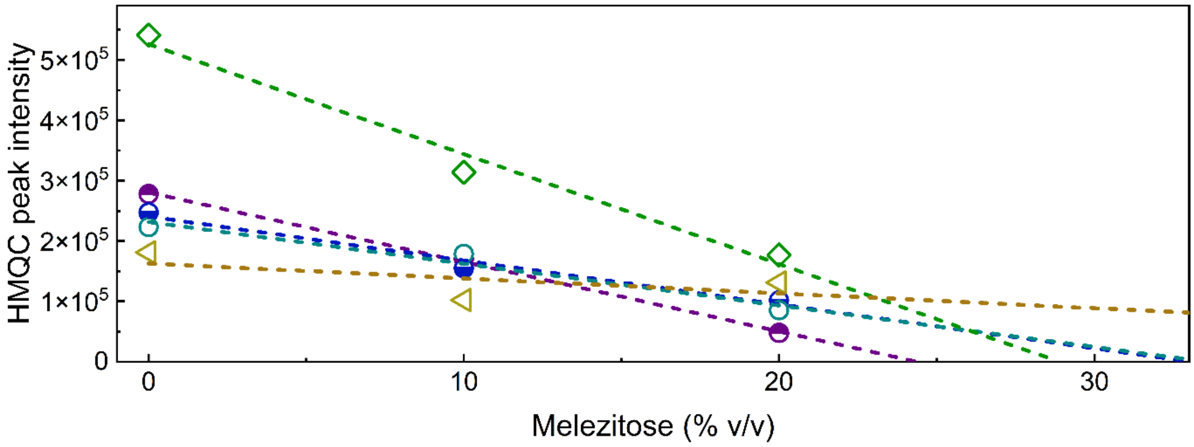

Fig. S9 HMQC peak intensities of the GA-Z residues Ile16$^{(C\gamma2\text{-}H\gamma2)}$ (◓), Leu28$^{(C\gamma\text{-}H\gamma)}$ (◒), Leu34$^{(C\gamma\text{-}H\gamma)}$ (○), Glu47$^{(C\beta\text{-}H\beta)}$ (◇), and Lys49$^{(C\gamma\text{-}H\gamma)}$ (◁) in 0-30% v/v sucrose analyzed at 37°C. The slope equations for the residues are I=-11504*X+280528 (---), I=-7281.9*X+240772 (---), I= 6901.1*X+231509 (---), I=-18220*X+526229 (---), and I=-2470.5*X+162949 (---). All samples contained 9 mg/mL GA-Z in PBS buffer (25 mM sodium phosphate, 125 mM NaCl, pH 7.0)

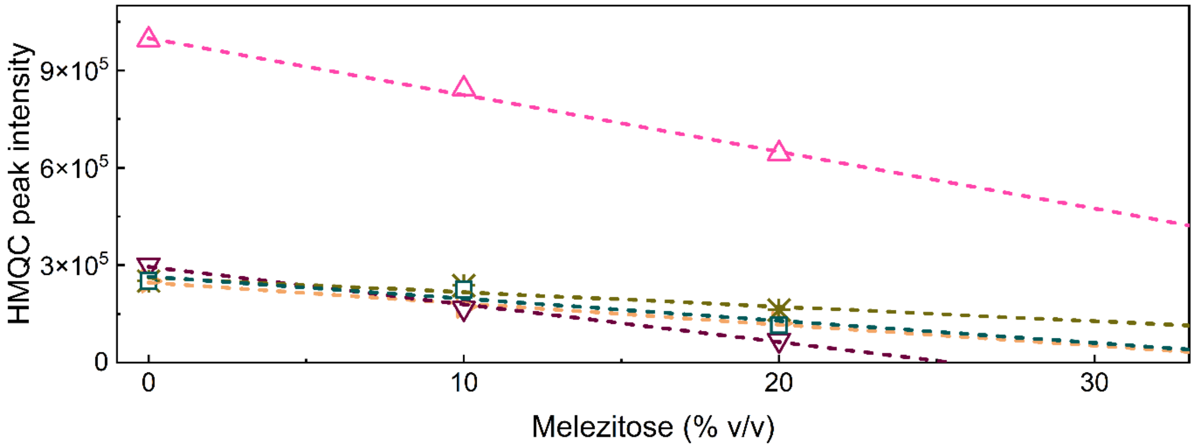

Fig. S10 HMQC peak intensities of the GA-Z residues Leu74$^{(C\delta2\text{-}H\delta2)}$ (▷), Lys84$^{(C\delta\text{-}H\delta)}$ (△), Ile103$^{(C\gamma2\text{-}H\gamma2)}$ (▽), Leu104$^{(C\delta2\text{-}H\delta2)}$ (*), and Leu107$^{(C\delta2\text{-}H\delta2)}$ (□) in 0-30% v/v sucrose analyzed at 37°C. The slope equations for the residues are I=-6489.9*X+246995 (---), I=-17509*X+1000000 (---), I=-11583*X+295216 (---), I=-4470.1*X+261794 (---), and I=-6809.8*X+265276 (---). All samples contained 9 mg/mL GA-Z in PBS buffer (25 mM sodium phosphate, 125 mM NaCl, pH 7.0)

Table S1. HMQC peak shifts of GA-Z residues in 0-30% v/v fructose, sucrose, and melezitose analyzed at 37°C. All samples contained 9 mg/mL GA-Z in PBS buffer (25 mM sodium phosphate, 125 mM NaCl, pH 7.0)

	**0% v/v**	**10% v/v**	**20% v/v**	**30% v/v**
**Fructose**				
Ile16-^{13}Cγ2 (ppm)	18.385	18.398	18.274	
Ile16-^{1}Hγ2 (ppm)	0.918	0.917	0.910	
Leu28-^{13}Cγ (ppm)	27.448	27.427	27.480	
Leu28-^{1}Hγ (ppm)	2.039	2.040	2.053	
Leu34-^{13}Cγ (ppm)	27.081	27.049	26.809	
Leu34-^{1}Hγ (ppm)	1.611	1.610	1.592	
Glu47-^{13}Cβ (ppm)	29.649	29.542	29.497	
Glu47-^{1}Hβ (ppm)	2.082	2.084	2.068	
Lys49-^{13}Cγ (ppm)	24.588	24.587	24.444	
Lys49-^{1}Hγ (ppm)	1.226	1.206	1.164	
Leu74-^{13}Cδ2 (ppm)	22.945	22.848	22.787	
Leu74-^{1}Hδ2 (ppm)	0.552	0.550	0.528	
Lys84-^{13}Cδ (ppm)	29.163	29.076	29.024	
Lys84-^{1}Hδ (ppm)	1.703	1.707	1.703	
Ile103-^{13}Cγ2 (ppm)	17.656	17.509	17.449	
Ile103-^{1}Hγ2 (ppm)	0.785	0.777	0.770	
Leu104-^{13}Cδ2 (ppm)	22.991	22.956	22.887	
Leu104-^{1}Hδ2 (ppm)	0.361	0.353	0.349	
Leu107-^{13}Cδ2 (ppm)	23.342	23.321	23.255	
Leu107-^{1}Hδ2 (ppm)	0.637	0.622	0.615	
**Sucrose**				
Ile16-^{13}Cγ2 (ppm)	18.385	18.410	18.035	
Ile16-^{1}Hγ2 (ppm)	0.918	0.915	0.891	
Leu28-^{13}Cγ (ppm)	27.448	27.479	27.474	
Leu28-^{1}Hγ (ppm)	2.039	2.048	2.051	
Leu34-^{13}Cγ (ppm)	27.081	27.022	27.004	
Leu34-^{1}Hγ (ppm)	1.611	1.612	1.617	
Glu47-^{13}Cβ (ppm)	29.649	29.549	29.464	
Glu47-^{1}Hβ (ppm)	2.082	2.075	2.082	
Lys49-^{13}Cγ (ppm)	24.588	24.547	24.500	24.459

Lys49-^{1}Hγ (ppm)	1.226	1.201	1.190	1.169
Leu74-^{13}Cδ2 (ppm)	22.945	22.856	22.907	
Leu74-^{1}Hδ2 (ppm)	0.552	0.547	0.554	
Lys84-^{13}Cδ (ppm)	29.163	29.128	29.069	28.942
Lys84-^{1}Hδ (ppm)	1.703	1.699	1.696	1.702
Ile103-^{13}Cγ2 (ppm)	17.656	17.477	17.345	
Ile103-^{1}Hγ2 (ppm)	0.785	0.785	0.768	
Leu104-^{13}Cδ2 (ppm)	22.991	22.966	22.871	22.866
Leu104-^{1}Hδ2 (ppm)	0.361	0.354	0.350	0.335
Leu107-^{13}Cδ2 (ppm)	23.342	23.326	23.292	23.277
Leu107-^{1}Hδ2 (ppm)	0.637	0.621	0.611	0.597
**Melezitose**				
Ile16-^{13}Cγ2 (ppm)	18.385	18.398	18.38	
Ile16-^{1}Hγ2 (ppm)	0.918	0.921	0.918	
Leu28-^{13}Cγ (ppm)	27.448	27.461	27.386	
Leu28-^{1}Hγ (ppm)	2.039	2.033	2.031	
Leu34-^{13}Cγ (ppm)	27.081	27.034	26.986	
Leu34-^{1}Hγ (ppm)	1.611	1.630	1.624	
Glu47-^{13}Cβ (ppm)	29.649	29.574	29.517	
Glu47-^{1}Hβ (ppm)	2.082	2.084	2.093	
Lys49-^{13}Cγ (ppm)	24.588	24.58	24.509	
Lys49-^{1}Hγ (ppm)	1.226	1.200	1.197	
Leu74-^{13}Cδ2 (ppm)	22.945	22.944	22.827	
Leu74-^{1}Hδ2 (ppm)	0.552	0.553	0.541	
Lys84-^{13}Cδ (ppm)	29.163	29.145	29.055	
Lys84-^{1}Hδ (ppm)	1.703	1.701	1.706	
Ile103-^{13}Cγ2 (ppm)	17.656	17.572	17.42	
Ile103-^{1}Hγ2 (ppm)	0.785	0.788	0.794	
Leu104-^{13}Cδ2 (ppm)	22.991	22.997	22.944	
Leu104-^{1}Hδ2 (ppm)	0.361	0.359	0.357	
Leu107-^{13}Cδ2 (ppm)	23.342	23.329	23.288	
Leu107-^{1}Hδ2 (ppm)	0.637	0.635	0.614	

Table S2. HMQC peak shifts of GA-Z residues in 0-10.9 mM DDM and 0-0.8 mM polysorbate 80 analyzed at 37°C. All samples contained 9 mg/mL GA-Z in PBS buffer (25 mM sodium phosphate, 125 mM NaCl, pH 7.0)

**DDM**	**0 mM**	**1.1 mM**	**5.5 mM**	**10.9 mM**
Lys4-^{13}Cδ (ppm)	29.184	29.202	29.188	29.169
Lys4-^{1}Hδ (ppm)	1.586	1.587	1.597	1.601
Ile16-^{13}Cγ2 (ppm)	18.385	18.407	18.482	
Ile16-^{1}Hγ2 (ppm)	0.918	0.913	0.925	
Leu19-^{13}Cδ2 (ppm)	24.147	23.976		
Leu19-^{1}Hδ2 (ppm)	0.636	0.667		
Thr23-^{13}Cδ (ppm)	21.978	22.010	21.990	21.982
Thr23-^{13}Hδ2 (ppm)	1.290	1.281	1.274	1.267
Leu28-^{13}Cγ (ppm)	27.448	27.515	27.487	27.434
Leu28-^{1}Hγ (ppm)	2.039	2.042	2.036	2.027
Leu34-^{13}Cγ (ppm)	27.081	27.105	27.082	27.066
Leu34-^{1}Hγ (ppm)	1.611	1.632	1.636	1.638
Glu47-^{13}Cβ (ppm)	29.649	29.660	29.711	29.899
Glu47-^{1}Hβ (ppm)	2.082	2.070	2.069	2.070
Lys49-^{13}Cγ (ppm)	24.588	24.638	24.630	
Lys49-^{1}Hγ (ppm)	1.226	1.226	1.208	
Leu74-^{13}Cδ2 (ppm)	22.945	23.044	22.973	22.951
Leu74-^{1}Hδ2 (ppm)	0.552	0.568	0.576	0.546
Lys84-^{13}Cδ (ppm)	29.163	29.628	29.230	29.218
Lys84-^{1}Hδ (ppm)	1.703	1.690	1.690	1.688
Lys91-^{13}Cδ (ppm)	24.606	24.657	24.682	
Lys91-^{1}Hδ (ppm)	1.143	1.157	1.165	
Thr92-^{13}Cδ (ppm)	21.940	22.010	21.935	21.940
Thr92-^{13}Hδ2 (ppm)	1.235	1.281	1.235	1.236
Ile103-^{13}Cγ2 (ppm)	17.656	17.619	17.683	17.681
Ile103-^{1}Hγ2 (ppm)	0.785	0.780	0.796	0.801
Leu104-^{13}Cδ2 (ppm)	22.991	22.998	22.987	22.956
Leu104-^{1}Hδ2 (ppm)	0.361	0.364	0.364	0.367
Leu107-^{13}Cδ2 (ppm)	23.342	23.407	23.138	23.383
Leu107-^{1}Hδ2 (ppm)	0.637	0.641	0.647	0.626
**Polysorbate 80**	**0 mM**	**0.1 mM**	**0.4 mM**	**0.8 mM**
Lys4-^{13}Cδ (ppm)	29.184	29.203	29.204	29.194

Lys4-^{1}Hδ (ppm)	1.586	1.586	1.585	1.586
Ile16-^{13}Cγ2 (ppm)	18.385	18.408	18.398	18.407
Ile16-^{1}Hγ2 (ppm)	0.918	0.920	0.919	0.920
Leu19-^{13}Cδ2 (ppm)	24.147	24.261	24.085	24.149
Leu19-^{1}Hδ2 (ppm)	0.636	0.644	0.648	0.647
Thr23-^{13}Cδ (ppm)	21.978	21.999	21.99	21.996
Thr23-^{13}Hδ2 (ppm)	1.290	1.290	1.290	1.290
Leu28-^{13}Cγ (ppm)	27.448	27.51	27.455	27.465
Leu28-^{1}Hγ (ppm)	2.039	2.046	2.030	2.032
Leu34-^{13}Cγ (ppm)	27.081	27.116	27.058	27.085
Leu34-^{1}Hγ (ppm)	1.611	1.604	1.626	1.622
Glu47-^{13}Cβ (ppm)	29.649	29.684	29.69	29.682
Glu47-^{1}Hβ (ppm)	2.082	2.085	2.082	2.081
Lys49-^{13}Cγ (ppm)	24.588	24.644	24.654	24.629
Lys49-^{1}Hγ (ppm)	1.226	1.235	1.218	1.233
Leu74-^{13}Cδ2 (ppm)	22.945	22.946	22.972	22.980
Leu74-^{1}Hδ2 (ppm)	0.552	0.556	0.557	0.564
Lys84-^{13}Cδ (ppm)	29.163	29.232	29.274	29.264
Lys84-^{1}Hδ (ppm)	1.703	1.702	1.690	1.692
Lys91-^{13}Cδ (ppm)	24.606	24.65	24.646	24.556
Lys91-^{1}Hδ (ppm)	1.143	1.146	1.160	1.156
Thr92-^{13}Cδ (ppm)	21.940	21.939	21.94	21.940
Thr92-^{13}Hδ2 (ppm)	1.235	1.235	1.235	1.235
Ile103-^{13}Cγ2 (ppm)	17.656	17.585	17.593	17.583
Ile103-^{1}Hγ2 (ppm)	0.785	0.790	0.787	0.786
Leu104-^{13}Cδ2 (ppm)	22.991	23.057	23.011	23.074
Leu104-^{1}Hδ2 (ppm)	0.361	0.363	0.359	0.361
Leu107-^{13}Cδ2 (ppm)	23.342	23.342	23.461	23.433
Leu107-^{1}Hδ2 (ppm)	0.637	0.642	0.643	0.642

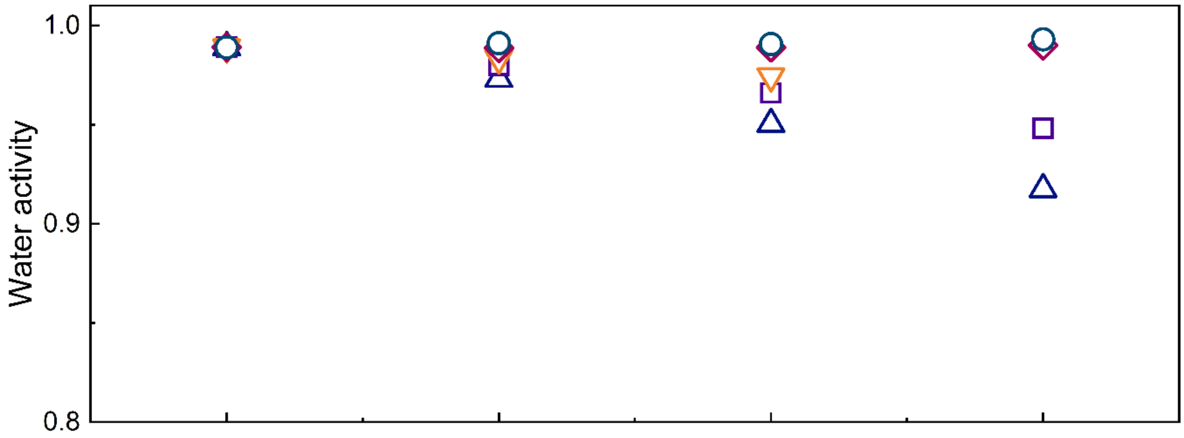

Fig. S11 The water activity of the stability samples containing 0, 10, 20, and 30% v/v fructose (△), sucrose (□), and melezitose (▽), 1.1, 5.5, and 10.9 mM DDM (◇), and 0.1, 0.4, and 0.8 mM polysorbate 80 (○) measured at 20°C. All samples contained 9 mg/mL GA-Z in PBS buffer (25 mM sodium phosphate, 125 mM NaCl, pH 7.0)
